# Trichostatin A suppresses hearing loss by reducing oxidative stress and inflammation in an Alport syndrome model

**DOI:** 10.1371/journal.pone.0316033

**Published:** 2025-02-05

**Authors:** Yoon Seok Nam, Eun-Ji Gi, Yoo-Seung Ko, Sungsu Lee, Hyong-Ho Cho

**Affiliations:** 1 Department of Otolaryngology-Head and Neck Surgery, Chonnam National University Medical School and Chonnam National University Hospital, Gwangju, Republic of Korea; 2 Department of Biomedical Science, College of Medicine, Chonnam National University Graduate School, BK21 PLUS Center for Creative Biomedical Scientists at Chonnam National University, Gwangju, Republic of Korea; Kyoto Prefectural University of Medicine, JAPAN

## Abstract

Alport syndrome (AS) is a genetic disorder marked by mutations in type IV collagen, leading to kidney glomerular dysfunction. AS also involves the cochlea, causing late-onset progressive hearing loss. Currently, there are no therapeutic drugs to protect hearing from AS. HDAC inhibitors (HDACis) are chemical compounds that block the activity of histone deacetylase and are known to exert diverse biologic effects. We investigated the effect of Trichostatin A (TSA), an HDACi, to assess its potential to inhibit hearing deterioration in AS. *Col4a3* knockout (KO) mice were treated with TSA at 3 weeks of age, and hearing levels were measured using auditory brainstem response (ABR). The results demonstrate that TSA significantly protects the hearing of KO mice compared to the untreated group. The TSA-treated group exhibited a reduction in the levels of oxidative stress markers 4-Hydroxynonenal and 3-Nitrotyrosine, along with a decrease in inflammatory cytokines, in both the mouse cochlea and in vitro HEI-OC1 (House Ear Institute-Organ of Corti 1) cell and HEK (Human Embryonic Kidney)293T cells. AS demonstrated a thickening in the stria vascular vessels, a phenomenon that TSA attenuated. Col4α3 deficiency showed down-regulation of Hemeoxygenase-1 (HO-1), a key anti-inflammatory molecule. TSA treatment induced HO-1 signaling, which contributed to the inhibition of oxidative stress and inflammatory cytokines. These findings suggest that TSA represents a promising candidate molecule for mitigating the progression of hearing loss in AS.

## Introduction

Sensorineural hearing loss is one of the most prevalent sensory disorders. The genetic cause of sensorineural hearing loss is rapidly being revealed by the advancements in NGS. Recently, there has been extensive application of gene therapy to replace or correct missing or defective genes in genetic hearing loss. Some gene defects, such as *OTOF* (Otoferlin), *TMC1* (Transmembrane channel-like protein 1), *KCNQ4* (Potassium voltage-gated channel subfamily Q 4), *VGLUT3* (Vesicular glutamate transporter 3), etc., have shown successful hearing preservation by this treatment [1–5]. However, it is important to perform these gene therapies at an early stage. Researchers conducted most of the studies on P1-P3 neonatal mice immediately after birth [6]. Performing the intervention at a later stage, when hearing deterioration had advanced, resulted in a low or no protective effect. So, this gene therapy works better for GHL that starts late and gets worse over time than for congenital hearing loss that starts early. Further, a method to inhibit the progression of the hearing loss may increase the effective time window of GHL gene therapy.

One of the late-onset progressive GHLs is AS. It can also cause serious health problems like progressive renal failure, sensorineural hearing loss, and eye disorder. The condition is mostly passed down through X-linked inheritance, with changes in the *COL4A5* gene being responsible for about 85% of cases [7]. Some cases have shown autosomal recessive and autosomal dominant patterns, which suggests that the *COL4A3* and *COL4A4* genes play a role in the development of the disease [8]. The major implication associated with AS on the morbidity and mortality of patients is ESRD [9]. Hearing loss in AS usually starts in the second decade of life and slowly progresses thereafter [10]. So far, there is no pharmaceutical therapy to protect against the hearing loss of AS.

HDACis are a promising class of epigenetic modulators that have shown considerable therapeutic potential in the treatment of various malignant diseases [11]. HDACi control gene expression by changing the acetylation status of protein substrates. This has an effect on many cellular processes, including the progression of the cell cycle, cell death, and differentiation [12]. Recent evidence suggests that HDACi may be a promising therapeutic agent in the context of hearing loss. Reports suggest that class I/II HDACi, SAHA, or valproic acid could potentially alleviate hearing loss [13, 14]. Another HDACi, TSA, was reported to be effective on spinal muscular atrophy and Alzheimer’s disease [15, 16]. Putting these together, we investigated the protective effects of TSA on AS’s auditory function and analyzed the feasibility of using an HDACi to modulate late-onset GHL.

## Results

### 1. TSA protects hearing in Col4α3 knockout mouse

**Fig 1:**
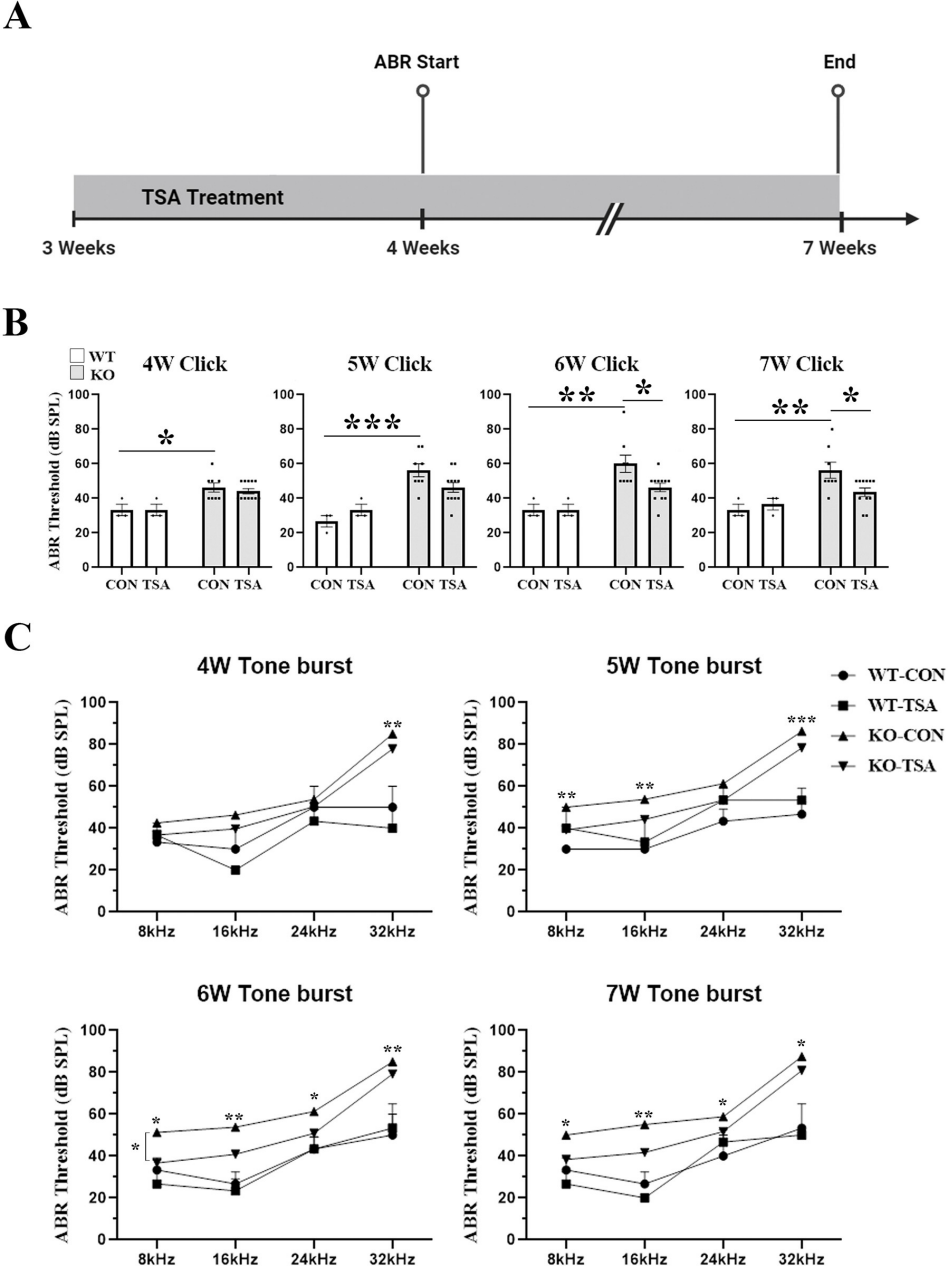
Systemic injection of TSA reduces hearing loss in *Col4a3* KO mice. (A) Experimental timeline. TSA was intraperitoneally injected into the WT and *Col4a3* KO mice. Mice, at the age of 3-weeks, were subjected to TSA treatment until 7 weeks. The auditory brainstem response (ABR) was evaluated from the 4-week to the 7-week using click and tone burst stimuli. (B) TSA attenuated *Col4a3* KO-induced hearing loss, as shown by ABR click results. Data are presented as mean ± S.E.M. (standard error of the mean). *P < 0.05, **P < 0.01, ***P < 0.001 based on ANOVA with Tukey’s post hoc test (n = 12). (C) The tone burst results showed a significant decrease at 8, 16, 24, and 32 kHz in TSA-treated *Col4a3* KO mice at 6–7 weeks. *P < 0.05, **P < 0.01, ***P < 0.001 based on ANOVA with Tukey’s post hoc test (n = 12).

The *Col4a3* KO mice exhibited signs of deafness prior to reaching 4 weeks of age. Therefore, TSA treatment (10 mg/kg body weight, once daily) was administered by intraperitoneal (IP) injection to WT and *Col4a3* KO mice from 3 weeks to 7 weeks (28 days) of age (Fig 1A). In this period, there were no significant changes in body weight in either the WT or *Col4a3* KO mice, regardless of whether they were in the control or TSA treatment group (S1 Fig). However, the *Col4a3* KO mice exhibited a significant alteration in their hearing sensitivity, whereby there was a gradual increase in the click auditory threshold of *Col4a3* KO mice beginning at week 4, which eventually reached a plateau of approximately 60 ± 5 dB SPL when they were 6 weeks old. In the *Col4a3* KO mice, the ABR threshold was significantly preserved in the group that received TSA treatment (43 ± 2 dB SPL) at 7 weeks of age, whereas the threshold in the control group (vehicle, 10% Kolliphor, only injected) was 56 ± 5 dB SPL. Although the TSA treatment only significantly attenuated the tone burst ABR effect at 8 kHz in 6-week-old *Col4a3* KO mice, the overall improvement in hearing sensitivity in these mice was observed from 4 to 7 weeks of age, which suggests that TSA treatment provides a beneficial effect on hearing function in this mouse model (Fig 1C). The Click ABR thresholds in the wild-type mice receiving TSA treatment between 4 and 7 weeks were similar to the control group and showed no harmful effects, with values of 36 ± 3 dB SPL and 33 ± 3 dB SPL, respectively (Fig 1B).

### 2. TSA attenuates the stria vascularis basement membrane thickening in *Col4a3* KO mice

**Fig 2:**
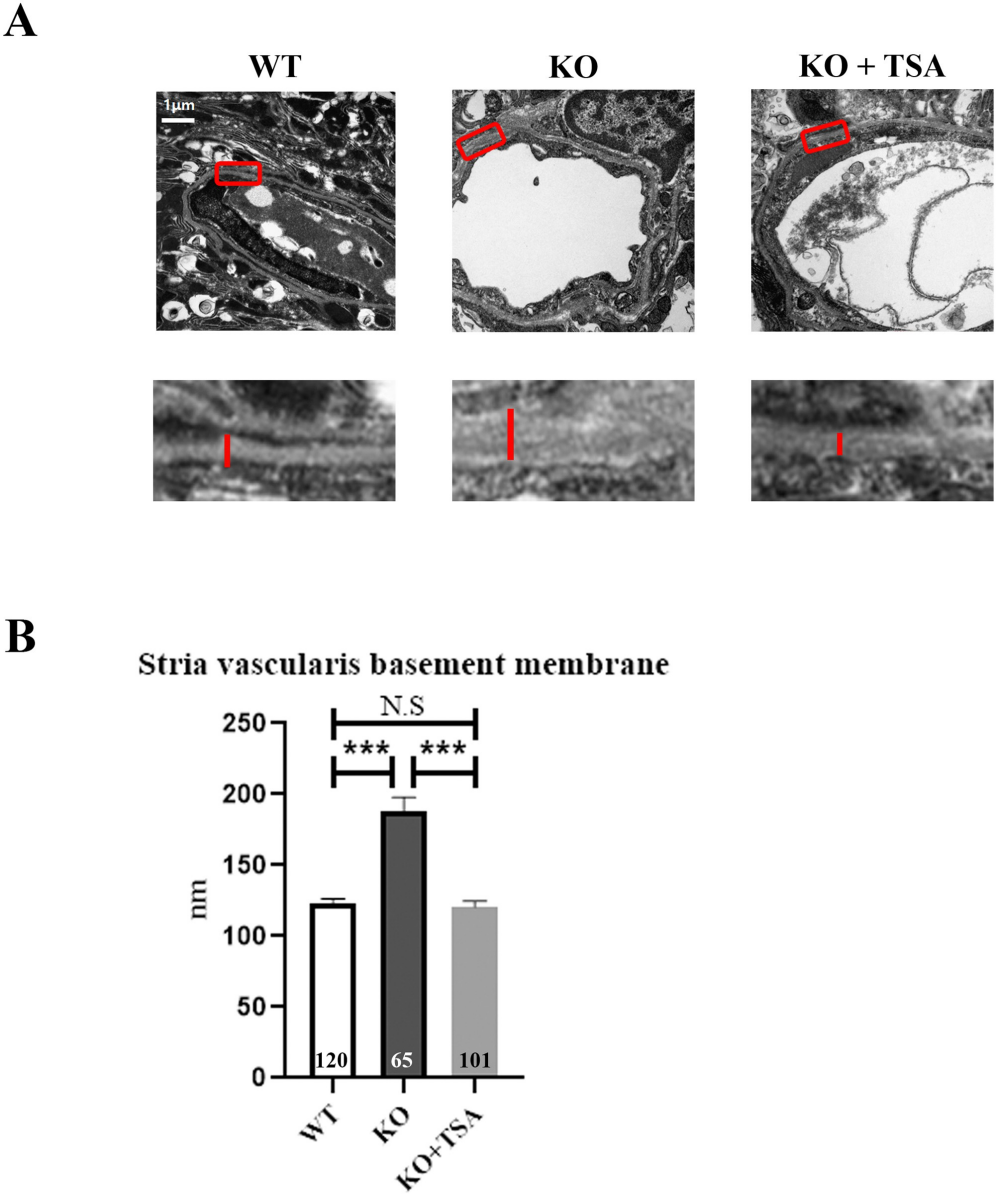
TSA attenuates *Col4a3* KO-induced stria vascularis basement membrane thickening. (A) Transmission electron microscopy (TEM) shows a thickened basement membrane of the stria vascularis in *Col4a3* KO mice, a pathology that is reduced by TSA treatment. Insets (red square) are shown at a higher magnification on the bottom. On the bottom lane, red straight lines illustrate the basement membrane. (B) Quantification results for the basement membrane thickness in the stria vascularis. Data are presented as mean ± S.E.M. *P < 0.05, **P < 0.01, ***P < 0.001 based on ANOVA with Tukey’s post hoc test. Numerals in bar graphs represent the number of samples.

Hearing loss in patients with AS is primarily caused by abnormalities in the structure of the cochlea. There is a defect in the proteins that form the basement membrane, which is the thin layer of tissue that supports the cells in the body, including the cells in the cochlea. This defect can cause the basement membrane to thicken and split, which can damage the hair cells and the basilar membrane and disrupt the transmission of sound waves to the brain. In this study, we used TEM to evaluate the SV basement membrane. Through meticulous observations of the SV basement membrane, we found that there was a noticeable thickening in the basement membrane of the *Col4a3* KO (Fig 2A). Conversely, TSA treatment alleviated the basement membrane thickening in the *Col4a3* KO (Fig 2B).

### 3. TSA alleviates inflammatory cytokine production induced by Col4α3 deficiency

**Fig 3:**
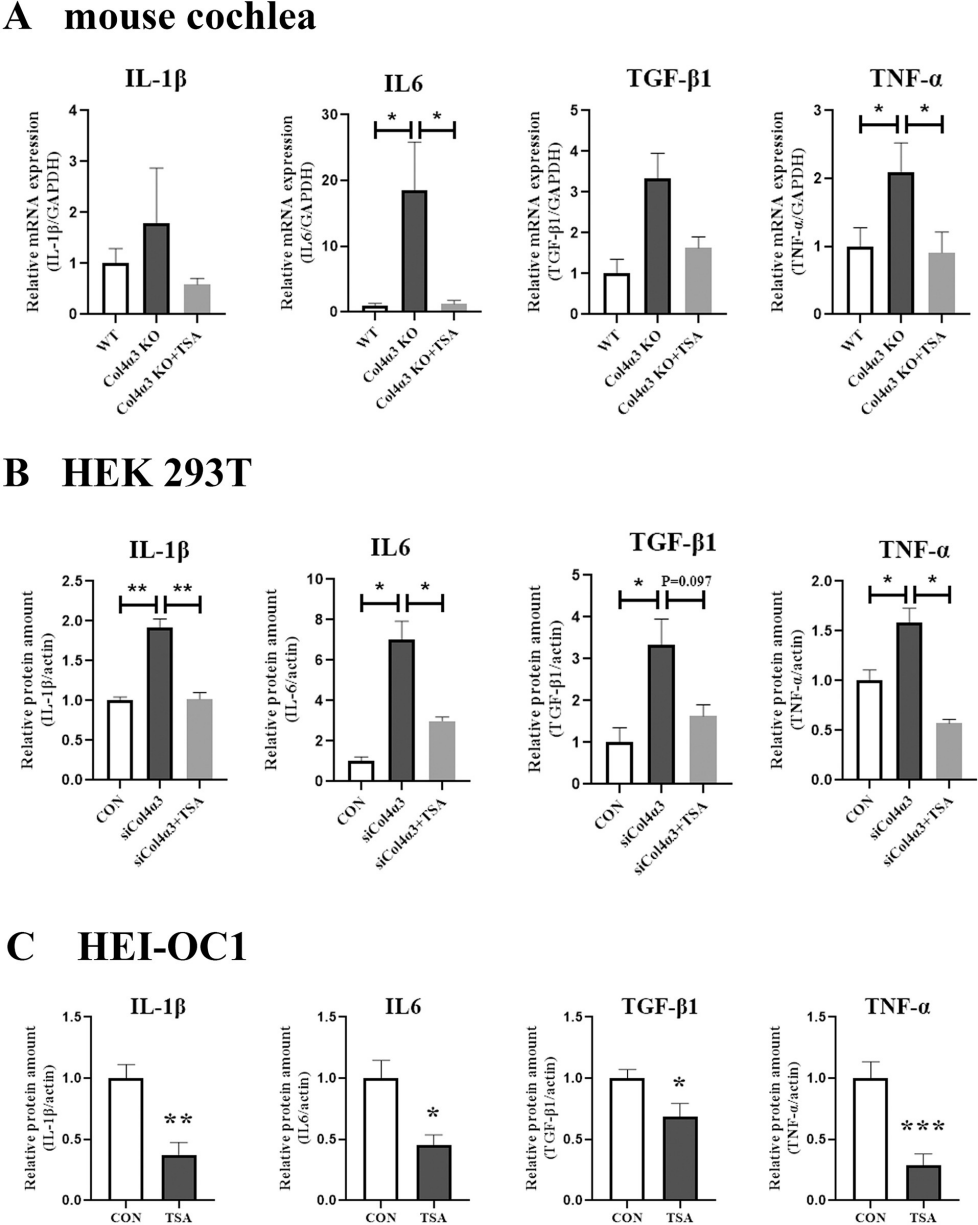
TSA alleviates the induction of inflammatory cytokines in *Col4a3* KO mice. (A) The mRNA level of proinflammatory cytokines IL-1β and TGF-β1 was higher in the cochlea of *Col4a3* KO mice, while IL-6 and TNF-α were significantly increased compared to wild-type (WT) mice. This effect was attenuated by TSA treatment (n = 7). (B) The protein level of IL-6, IL1β, TNF-α, and TGF-β1 was all significantly increased in HEK293T cells following transient transfection of siCol4α3. This effect was attenuated by TSA treatment (n = 3). (C) TSA treatment significantly decreased IL-6, IL1β, TNF-α, and TGF-β1 protein levels in HEI-OC1 cells (n = 7). Data are presented as mean ± SEM. *P < 0.05, **P < 0.01, ***P < 0.001 based on ANOVA with Tukey’s post hoc test.

Next, the mRNA expressions of the proinflammatory cytokines IL6, IL-1β, TNF-α, and TGF-β1 were higher in the cochlea of the *Col4a3* KO mice than in the WT mice. TSA treatment in *Col4a3* KO mice attenuated the mRNA expressions of IL-1β and TGF-β1 and significantly decreased the levels of the IL6 and TNF-α proinflammatory cytokines (Fig 3A). When COL4α3 was suppressed in HEK293T cells using siRNA, the protein expression levels of the proinflammatory cytokines were significantly higher than in the control group. These elevated levels of protein in the *COL4A3* knockdown group were attenuated by TSA treatment (Fig 3B). The HEI-OC1 cells displayed a significant decrease in the expression of the proinflammatory cytokine proteins following TSA treatment (Fig 3C). These data help to improve our understanding of how TSA treatment can protect against GHL in *Col4a3* KO mice.

### 4. TSA mitigates oxidative stress in the stria vascularis

**Fig 4:**
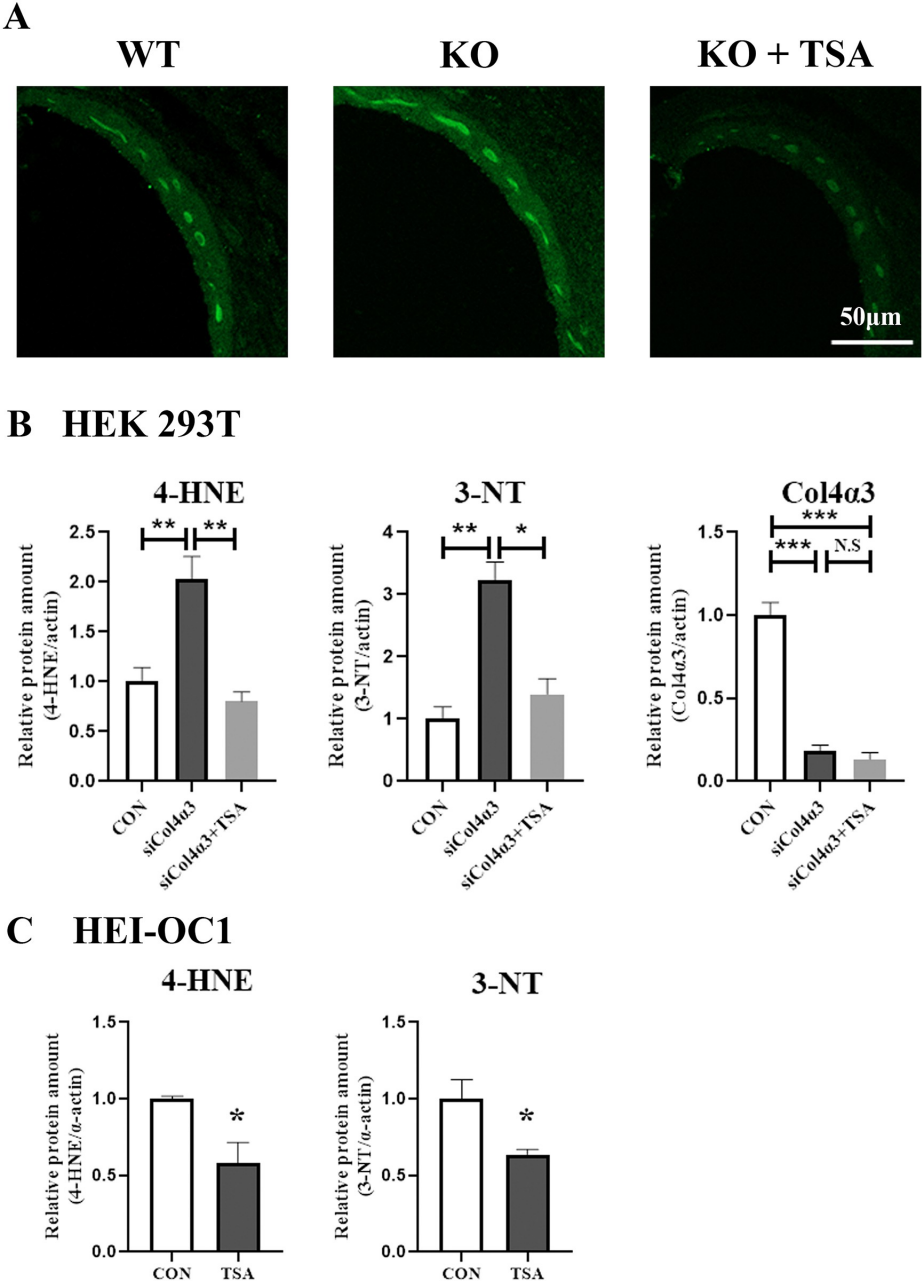
TSA inhibits *Col4a3* KO-induced oxidative stress in stria vascularis. (A) Representative image of SV mice. SV immunostaining was conducted using antibodies directed towards 4-HNE (green). (B) The protein expressions of 4-HNE and 3-NT were significantly increased in HEK293T cells after transient transfection with siCol4α3. This effect was attenuated by TSA treatment (n = 6). Data are presented as mean ± SEM. *P < 0.05, **P < 0.01, ***P < 0.001 based on ANOVA with Tukey’s post hoc test. (C) Treatment of HEI-OC1 cells with TSA resulted in reduced expressions of 4-HNE and 3-NT proteins. The quantification results showed that TSA treatment of HEI-OC1 cells reduced the protein expressions of 4-HNE and 3NT (n = 3). Data are presented as mean ± SEM. *P < 0.05, **P < 0.01, ***P < 0.001 based on ANOVA with Tukey’s post hoc test.

Two important and established markers of oxidative stress are 4-HNE and 3-NT, which are also known to be involved in apoptosis [17]. To determine whether 4-HNE in the cochlea is inhibited by TSA treatment, we performed immunostaining for 4-HNE and analyzed the results. The 4-HNE staining was more pronounced in the SV of *Col4a3* KO compared to WT mice. Notably, the staining was significantly reduced in the *Col4a3* KO mice treated with TSA (Fig 4A). Likewise, the expression of 4-HNE and 3-NT was significantly increased in the HEK293T cells following *COL4A3* knockdown. However, TSA treatment significantly reduced their expressions (Fig 4B). In addition, the protein expression of 4-HNE and 3-NT was significantly decreased in the HEI-OC1 cells following TSA treatment (Fig 4C). Therefore, we concluded that injecting TSA can effectively reduce Reactive oxygen species (ROS) production within the cochlea.

### 5. TSA attenuates KEAP1 and upregulates HO-1 expression in *Col4a3* deficiency

**Fig 5:**
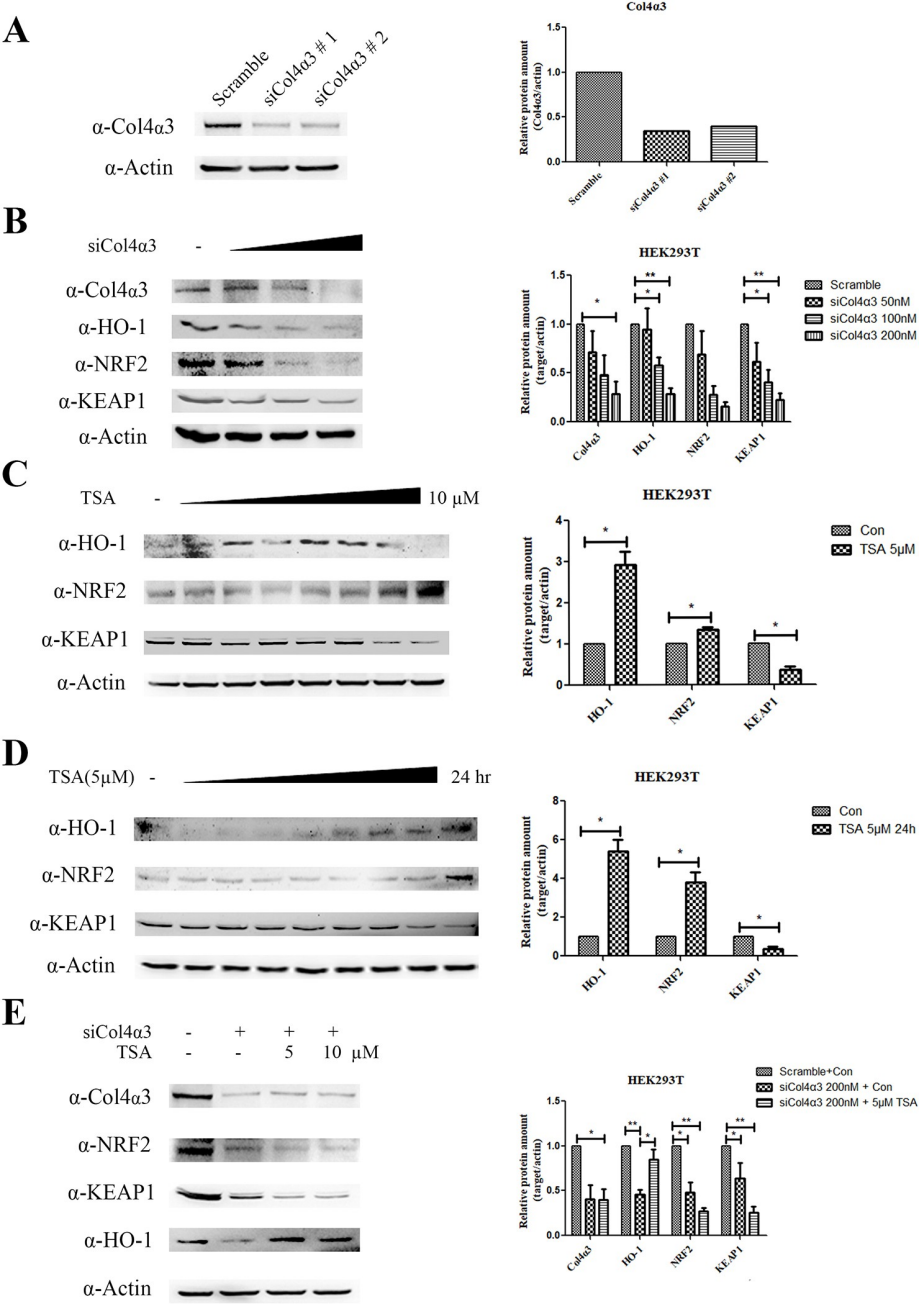
TSA induces HO-1 and NRF2 expression and inhibits KEAP1, while Col4α3 knockdown blocks NRF2 induction in vitro. (A) Transient transfection of siCol4α3 reduced Col4α3 protein expression in HEK293T cells. Quantification results. (B) Dose-dependent transient transfection of siCol4α3 reduced Col4α3, HO-1, NRF2, and KEAP1 protein expressions in HEK293T cells. Quantification results: * p < 0.05, ** p < 0.01, The number of samples is 3. (C) TSA induces dose-dependent NRF2 and HO-1 protein expressions and inhibits KEAP1 expression; error bars represent S.E.M. Quantification results: * p < 0.05, the number of samples is 3. Error bars represent S.E.M. (D) NRF2 and HO-1 increase protein expression in a time-dependent manner following 5 μM TSA treatment, whereas KEAP1 expression is inhibited. Quantification results. * p < 0.05, The number of samples is 3. Error bars represent S.E.M. (E) TSA recovers HO-1 expression following transient transfection of siCol4α3 and a reduction in Col4α3, NRF2, KEAP1, and HO-1 protein expressions in HEK293T cells. Quantification results: * p < 0.05, ** p < 0.01, The number of samples is 3. Error bars represent S.E.M.

**Fig 6:**
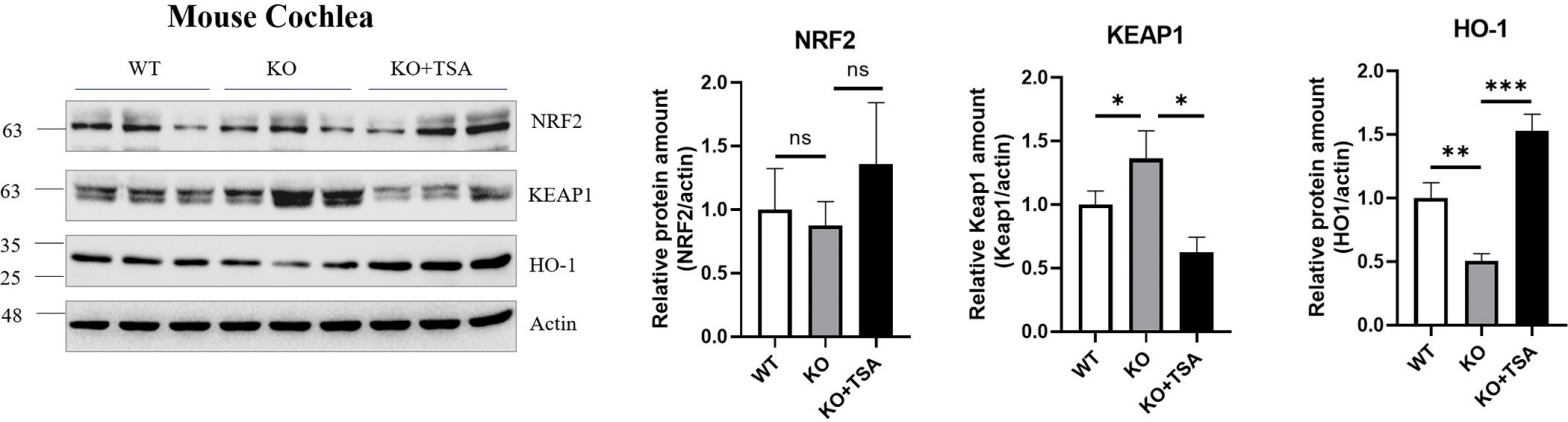
TSA induces HO-1 and inhibits KEAP1 in *Col4a3* KO cochlea in vivo. In the *Col4a3* knockout (KO) cochlea, NRF2 showed no significant change and was not affected by TSA treatment (p = 0.99 and p = 0.52). However, KEAP1 was significantly suppressed by TSA (p = 0.01). *Col4a3* KO led to a downregulation of HO-1 (p = 0.04), and TSA treatment effectively restored HO-1 levels in the cochlear tissue (p = 0.0002). Quantification results: * p < 0.05, *** p < 0.001. The number of samples is 3 for each group. ns, not significant. Error bars represent S.E.M.

To further explore the molecular mechanism of the TSA, we examined the NRF2, KEAP1, and HO-1 signaling pathways, which are well-known key factors in oxidative stress [18]. Initially, we assessed the regulation of TSA through an in vitro study using HEK293T cells. siRNA targeting *COL4A3* successfully suppressed COL4α3 protein levels (Fig 5A). In addition, our experiment showed that siCol4α3 decreased HO-1 and NRF2 signaling while decreasing *COL4A3* mRNA expression in a way that depended on the dose. In addition, the level of KEAP1 was decreased due to the inhibition of COL4α3 (Fig 5B). Upon dose-dependent TSA treatment, which is doses under 50 μM so that the dose remains under the IC_50_ value (S2 Fig), a reduction in KEAP1 level and induction of NRF2 and HO-1 were observed. However, treatment with a high dose (10 μM) of TSA did not maintain the induced levels of HO-1 (Fig 5C). Treatment with 5 μM TSA in a time-dependent manner inhibited KEAP1 and induced NRF2 and HO-1 (Fig 5D). Furthermore, siRNA suppression of COL4α3 protein expression disrupted the induction of NRF2 following TSA treatment. Surprisingly, in the reduced NRF2 state, HO-1 induction was still observed upon TSA treatment. This result demonstrated that TSA induces HO-1 expression, which contributes to the inhibition of oxidative stress and inflammatory cytokines (Fig 5E). We validated these results using in vivo cochlear samples. NRF2 level was slightly reduced in the *Col4α3* KO cochlea and showed a slight increase with TSA, though this was not statistically significant (Fig 6). However, KEAP1 level was suppressed by TSA. Moreover, HO-1 level, which was significantly reduced in the *Col4a3* KO cochlea, was restored with TSA treatment.

### 6. Schematic diagram of TSA attenuating hearing loss in *Col4a3* KO mice

**Fig 7:**
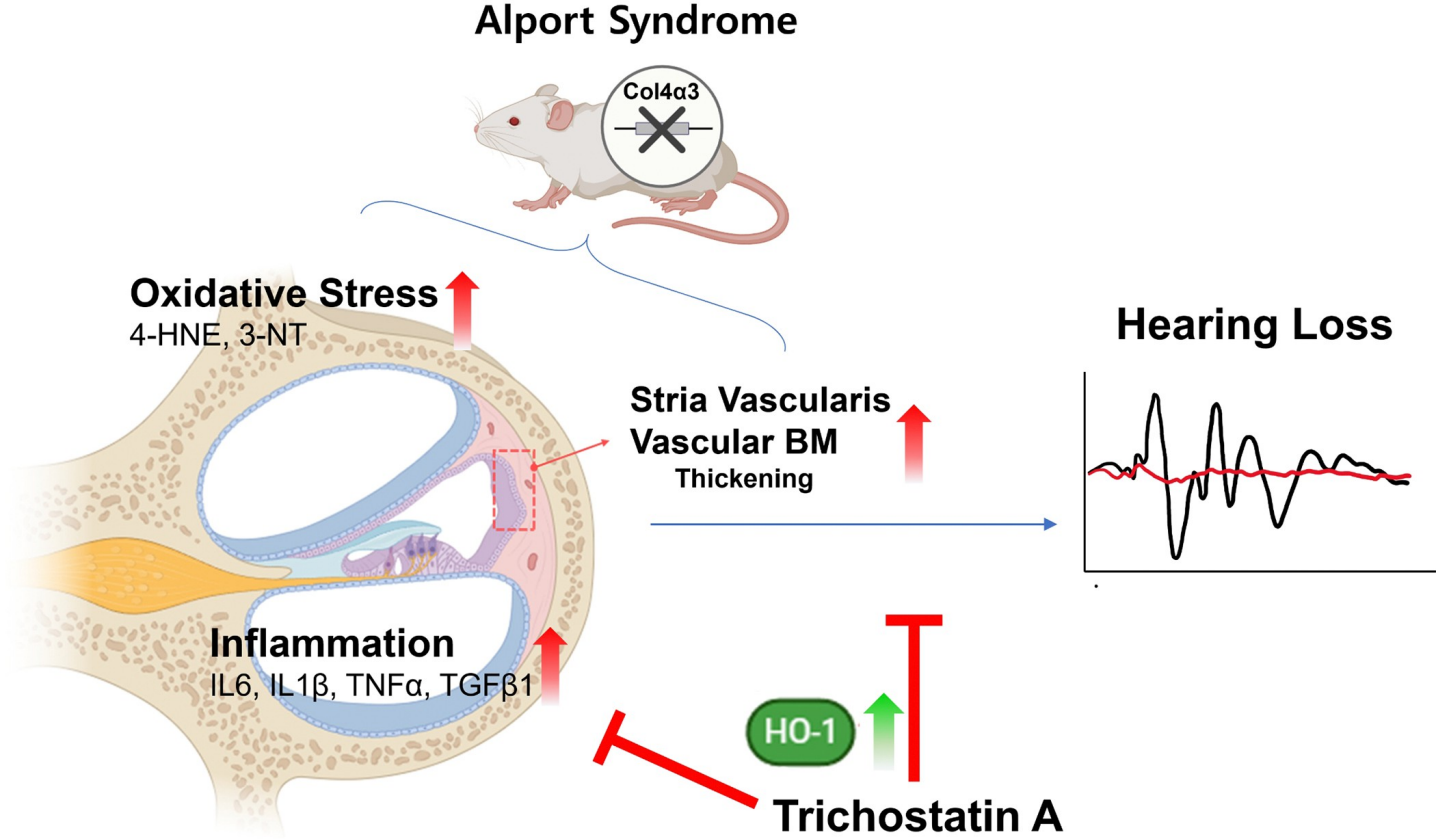
Schematic diagrams of TSA protection against progressive sensorineural hearing loss induced by *Col4a3* KO. Collagen type 4 α3 chain (*Col4a3*) knockout (KO) induces inflammation and oxidative stress in the cochlea. Basement membrane (BM) of the stria vascularis vessels is thickened which all leads to hearing impairment. Trichostatin A (TSA) increases the expression of HO-1. The upregulation of HO-1 inhibits proinflammatory gene activation, ROS gene production and reduces stria vascularis BM thickening.

TSA inhibits KEAP1 and increases HO-1 level. TSA induces HO-1, which inhibits proinflammatory genes and ROS production (Fig 7). A major problem in AS is an increase in inflammation and oxidative stress; therefore, this issue can be reduced by TSA treatment through HO-1 expression induction. Thus, TSA is a potential therapeutic agent for mitigating hearing deterioration in an AS model.

## Discussion

This is the first study to demonstrate that HDACi can protect AS-induced hearing loss. *Col4a3* KO decreases the level of HO-1, causing thickening of the stria vascularis basement membrane. An HDACi, TSA treatment reduces this thickening. In addition, TSA may prevent *Col4a3* KO-induced stria vascularis thickening and hearing loss by upregulating HO-1 level, which inhibits inflammatory cytokines and ROS production.

TSA, a class I/II HDACi [19], was identified as a natural derivative of dienohydroxamic acid from a fungal metabolite [20]. Research has shown that TSA has various therapeutic activities, including anti-diabetic and anti-cancer effects. It has also been found to improve motor function and survival rate in spinal muscular dystrophy and reduce Alzheimer’s pathology [15, 16]. Although TSA is mainly used in preclinical research, many other class I/II HDAC inhibitors, such as valproic acid, romidepsin, and vorinostat, are currently used in the clinic [21]. Recently, several studies reported protective effects of HDACi on hearing loss [13, 14]. Therefore, we wanted to investigate the effect of HDACi in AS, a model for late-onset progressive hearing loss.

Studies examining the cause of hearing loss associated with AS have provided insights into the underlying physiological mechanisms. Early research on the temporal bones of humans and mice mostly showed atrophy of stria vascularis and damage of inner and outer hair cells, as well as neural degeneration sometimes. In contrast, later studies described the presence of damage near the basilar membrane [22–25]. TSA treatment in our AS model demonstrated attenuation of stria vascularis basement membrane thickening. This physiological protection contributes to the reduction of hearing loss in *Col4a3* KO mice. This particular process, from a proximodistal perspective, may play a role in reducing inflammation and oxidative stress associated with basement membrane thickening in the stria vascularis.

An increase in the expression of inflammatory cytokine genes has been reported in AS models [26]. *Col4a3* KO mice exhibit increased inflammatory cytokine gene mRNA expression. Similarly, *Col4a3* knockdown cells show a significant increase in inflammatory protein expression. The TGFβ1, TNFα, IL-6, and IL-1β gene expressions in *Col4a3* KO mice are reduced by TSA treatment, as well as by in vitro *Col4a3* knockdown in HEK293T cells and HEI-OC1 cells. TSA treatment in various mouse models of different diseases has been shown to reduce inflammation [27]. Likewise, in AS experimental models, TSA treatment effectively reduces the associated increase in inflammation. Immunohistochemistry staining of the SV with 4-HNE indicated an increase in ROS due to *Col4a3* KO, which was reduced following TSA treatment. Moreover, these results demonstrate that, in vitro, HEK293T cells showed an increase in ROS production following siCol4α3 transient transfection; however, this increase was reduced by TSA treatment. In addition, HEI-OC1 cells showed reduced ROS gene expression due to TSA treatment.

NRF2 is a key player in controlling cellular responses that protect against oxidative stress [18]. NRF2 levels are modulated by KEAP1, which binds to NRF2 and facilitates its ubiquitination and degradation [28]. When oxidative stress occurs, KEAP1 experiences structural changes due to its interaction with any reactive species that the cell is exposed to. These structural changes lead to the dissociation of KEAP1 from NRF2, thereby allowing NRF2 to migrate freely into the nucleus [29]. This NRF2-mediated process activates antioxidant enzymes such as HO-1. Interestingly, there were some differences in NRF2 expression between HEK293T cells and *Col4a3* KO cochlea. In our in vitro HEK293T study, NRF2 level was reduced by *Col4a3*. However, in the cochlea, NRF2 level did not show a significant change. It is possible that instead of altering NRF2 expression levels, the localization of NRF2 or phosphorylated NRF2 may have shifted, similar to findings from a previous study [30]. The specific mechanisms of COL4α3 deficiency and NRF2 involvement might differ between the kidney and cochlea.

HO-1 is a protective factor with potent anti-inflammatory, antioxidant, and antiproliferative effects. HO-1, the inducible isoform, is expressed in various tissues and is upregulated by multiple stimuli [31]. Moreover, HO-1 is known to play a role as a mediator against noise-induced ototoxicity in HL [32]. TSA is known to induce NRF2 by inhibiting KEAP1, which leads to an increase in HO-1 [33, 34]. When we knock-downed *COL4A3*, TSA could not induce NRF2. Instead, HO-1 level increased as a result of the TSA treatment. These results suggest that TSA may be an effective drug for preventing ROS and inflammation resulting from *Col4a3* KO through an increase in HO-1 expression.

There are several limitations to our study. The target molecule for TSA is HDACi. All cells throughout our body express HDACi, and it is not specific to the cochlea. Studies have reported TSA’s anti-diabetic, anti-cancer, and anti-angiogenic effects [20, 35, 36]. Using TSA for treating AS’s hearing loss may influence these processes or related diseases. It is necessary to conduct further research on TSA through local cochlear drug delivery to reduce this off-target effect. In the current study, we did not find any toxicity in TSA systemic injection and mice gained normal body weight (S1 Fig). However, TSA itself needs validation of its safety and efficacy in human patients. Lastly, AS is one example of many GHLs. Each gene has its unique function in hearing and GHL has many different pathophysiologies. Thus, TSA treatment cannot be generalized to all GHLs.

Overall, hearing loss occurs in the AS mouse model with *Col4a3* KO through the induction of ROS and inflammation. Furthermore, AS causes the SV basement membrane to thicken and reduces NRF2 signaling. Treatment with TSA improves SV basement membrane thickening by reducing ROS and inflammation. Furthermore, TSA increases HO-1 signaling, which helps to alleviate inflammation. Therefore, TSA potentially serves as a therapeutic drug to prevent hearing loss progression in AS.

## Materials and methods

### Alport syndrome model and animal care

Type IV collagen alpha 3 chains (Col4α3) KO mice were purchased from Jackson laboratory, used as the AS model (Jackson laboratory, strain #002908). The use and care of the animals in this study were approved by the Institutional Animal Care and Use Committee at Chonnam National University Hospital (CNUHIACUC-21048). The mice were sheltered in a standard-conditioned vivarium with free access to food and water. Cages were changed every week, and food and water were replenished every three days. Mice were monitored daily for their health status and any signs of discomfort. All mice were healthy throughout the experimental period. The sample sizes for each experiment were carefully assessed and are provided in each figure. The animal experiments adhered strictly to the Guide for the Care and Use of Laboratory Animals of Chonnam National University Hospital and were reported according to ARRIVE guidelines (https://arriveguidelines.org).

### TSA treatment and cochlear tissue harvest

TSA (Cayman Chemical Company, 89730) was dissolved in 10% kolliphor in each desired concentration. For the in vivo study, a TSA dose of 10 mg/kg was used, consistent with previous reports [15, 16]. The treatment group was intraperitoneally injected with TSA from 3 weeks of age. The control group received the vehicle with only 10% kolliphor. Cochleae were harvested at 7 weeks, decalcified in ethylenediaminetetraacetic acid (EDTA), and cut and stained for immunofluorescence using 4-HNE and 3NT staining. Basement membrane thickness was checked using TEM.

### Auditory brainstem response (ABR) to evaluate animal hearing

We recorded the ABR using a 3RZ6 TDT system (Tucker-Davis Technologies, Alachua, FL 32615, USA), which provided stimuli ranging from clicks to tone bursts. Needle electrodes of 1.5 mm in length were inserted sub-dermally at the dorsal midline between the eyes (none inverting), at the scalp, and posterior to both pinnae. We tested various stimulus intensity levels in decreasing order at each frequency, from 90 to 20 dB of the visual ABR threshold. While performing ABR, mice were anesthetized with a cocktail of ketamine 80 mg/kg and xylazine 10 mg/kg, and they remained asleep during all ABR recordings. Hearing levels were measured by ABR from 4 weeks of age to 9 weeks, when homozygous KO mice began to die of end-stage renal failure.

### Immunohistochemistry

Mice were euthanized using a ketamine and xylazine cocktail (80 mg/kg and 10 mg/kg, respectively) prior to cochlea extraction. A small hole was created at the apex of each cochlea using a 0.5 cc syringe, and the cochlea was then perfused with phosphate-buffered saline (PBS), followed by 4% paraformaldehyde (PFA). The cochleae were subsequently immersed in 4% PFA for 1 hour with gentle rotation at 4°C. After rinsing twice with PBS, decalcification was performed using 0.12 mM EDTA for 1 hour with gentle rotation at 4°C. To expose the Organ of Corti, the surrounding bone and stria vascularis were dissected, and the tectorial membrane was removed. The cochlea was sectioned into three parts (apex, middle, and base), and the tissue samples were immersed in a blocking buffer for 1 hour at room temperature (RT), followed by overnight incubation with primary antibodies at 4°C. After three washes with 0.1% PBS-T (30 minutes per wash), the samples were incubated with secondary antibodies at room temperature for 2 hours. The samples were then washed three times with 0.1% PBS-T for 30 minutes each, stained with 4’,6-diamidino-2-phenylindole (DAPI) for 3 minutes, and rinsed in PBS for 30 minutes. A vector protection solution was used to mount the samples on glass slides, which were then examined using a laser scanning microscope (LSM 800, Carl Zeiss Microscopy GmbH, Promenade 10, 07745 Jena, Germany). The following antibodies were used: 4-HNE (#BS-6313R, Thermo Fisher Scientific Inc., Waltham, MA, USA) and 3-NT (AB61392, Abcam, Waltham, MA, USA).

### Antibodies

Primary antibodies used in this study were anti-Col4α3 (MBS9142287, MyBioSource, Inc., San Diego, CA 92195, USA), anti-3-nitrotyrosine (AB61392, Abcam, Waltham, MA, USA), anti-Actin (A2066, Sigma-Aldrich, Inc., St. Louis, MO, USA), anti-4 hydroxynonenal (#BS-6313R, Thermo Fisher Scientific Inc., Waltham, MA, USA), anti-HO-1 (#70081), anti-KEAP1 (#8047), and anti-NRF2 (#12721, Cell Signaling, Beverly, MA, USA). Anti-NRF2 (sc-365949) and anti-KEAP1 (sc-365626) antibodies were also purchased from Santa Cruz Biotechnology, Inc. (Santa Cruz, CA, USA). HRP-conjugated secondary antibodies were acquired from Thermo Fisher Scientific Inc. (Waltham, MA, USA), including goat anti-mouse-HRP (31430), and goat anti-rabbit-HRP (32460).

### TEM (transmission electron microscope)

Sample preparation and fixation: The samples were fixed using a 1% osmium tetroxide (OsO4) fixative solution. Fixation was conducted for an appropriate time (1 hour) before the sample was separated from the OsO4. The fixed sample underwent a dehydration process, whereby it was sequentially treated with increasing ethanol concentrations (50%, 70%, 80%, 90%, and 100%), with each concentration replaced every 10 minutes.

Embedding and polymerization with Epon mixture: To infiltrate the dehydrated sample with the Epon resin mixture, a series of Epon resin–ethanol mixtures were used with gradually increasing concentrations. This allows the resin to fully infiltrate the sample. The fully infiltrated sample was embedded in the Epon mixture, an electron microscopy block was formed, and then it underwent a heat polymerization process.

Ultra-thin sectioning: Using an ultramicrotome (PowerTome XL, Boeckeler Instruments, Inc, Tucson, AZ, USA), the electron microscopy block was trimmed into ultra-thin sections, approximately 80 nm in thickness, which were suitable for examination by electron microscopy.

Transmission electron microscopy (TEM) analysis: The prepared glass sections were placed on an electron microscope grid, which uses a high-resolution film with high electron accessibility. An electron microscope (JEM-1400, JEOL Ltd, Peabody, MA, USA) was used to capture images and collect data at various electron speed settings (60 kV).

### MTT assay for cell viability assessment

These distinct cell types were subjected to MTT (3-(4,5-di**methylthiazol**-2-yl)-2,5-diphenyl**tetrazolium** bromide) assays after the completion of each treatment regimen. The cells were collected in sterile Eppendorf tubes, and an MTT assay was performed 24 hours after all treatments, in accordance with the manufacturer’s protocol. Absorbance was measured using a Spectra Max 5 Plate Reader and Soft Max Pro 5.2 (Molecular Devices, Sunnyvale, CA, USA), and the average optical density (OD) was used to evaluate the viability against the OD of control cells, which was considered the fold change in the HEK293T cell line.

### Protein preparation

For Western blot, cell lysates were obtained using NP lysis buffer (1% Nonidet-P40, 50 mM Tris pH 8.0, 150 mM NaCl, 10 mM NaF, 1 mM Na3VO4, 5 mM EDTA, 1 mM EGTA, 1 mM PMSF, 1 mM DTT) supplemented with a protease inhibitor cocktail.

### In vitro cell culture and siRNA transfection

HEI-OC1 cells were cultured under permissive conditions (33°C). High-glucose Dulbecco’s modified Eagle’s medium (11965092, Gibco BRL, Gaithersburg, MD, USA) containing 10% fetal bovine serum (16000044, Gibco BRL, Gaithersburg, MD, USA) and 50 U/mL gamma interferon (Genzyme, Cambridge, Mass, USA) was used without antibiotics.

HEK293T cells were maintained according to the manufacturer’s instructions. The cells transfected with siRNA were incubated at 37°C for 48 h. For the knockdown of Col4a3 expression, two siRNA target sites were chosen from the human type IV collagen alpha 3 chains (COL4A3) mRNA sequence (GenBank accession number NM_000091.5), which was extracted from the NCBI Entrez nucleotide database. These target sites were also investigated using a BLAST search (National Center for Biotechnology Information) to confirm that the sites were specific to the human COL4A3. The 21-nucleotide sense and antisense RNA sequences are as follows:

COL4A3 siRNA #1,

5’- AGCAAGGGUUGUGUCUGUAUU-3’ (sense)

5’- UACAGACACAACCCUUGCUUU-3’ (antisense)

COL4A3 siRNA #2,

5’- CGGGUGAUAUGGGAAAGAAUU -3’ (sense)

5’- UUCUUUCCCAUAUCACCCGUU-3’ (antisense).

The negative control siRNA duplex was purchased from Bioneer company (Seoul, Korea), with the following sequences: 5’-CCUACGCCAAUUUCGUdTdT-3’ and 5’-ACGA AAUUGGUGGCGUAGGdTdT-3’. The siRNA duplexes were transiently transfected into the cells using Lipofectamine™ RNAiMAX, as described by the manufacturer (13778075, Thermo Fisher Scientific Inc., Waltham, MA, USA).

### RNA isolation and real-time polymerase chain reaction

Total RNA was isolated using TRIzol Reagent (15596026, Thermo Fisher Scientific) as described in the manufacturer’s protocol. DNase I (89836, Thermo Fisher Scientific) was used to remove residual DNA in RNA samples. RNA integrity was confirmed using a full-spectrum spectrophotometer (NanoDrop ND-1000, Technologies Inc., Wilmington, DE, USA) with absorbances measured at A260/A280 nm to determine the RNA amounts. First-strand complementary DNA (cDNA) was obtained by an RNA reverse-transcription cDNA synthesis kit (6110A, first-strand cDNA Synthesis kit; Takara Prime-ScriptTM, Japan), using the following cycle conditions: denaturation at 95°C for 10 minutes and 10 seconds, followed by annealing at 62°C for 20 seconds, and 72°C for 30 seconds, for a total of 60 cycles; the Taq Master Mix was used for RT-PCR (4444434, Bioscience, Germany). The 2-Ct technique was used to determine the expression levels, and the relative mRNA expression was adjusted to the glyceraldehyde 3-phosphate dehydrogenase expression (GAPDH). Each experiment was performed three times. The primers used for each gene are listed in Table 1 below:

**Table 1:** List of primers used for quantitative real-time PCR.

TGF-β1 forward	5ʹ- CAACAATTCCTGGCGTTACCTTGG-3ʹ
TGF-β1 reverse	5ʹ- GAAAGCCCTGTATTCCGTCTCCTT-3ʹ
IL-1β forward	5ʹ-GCTGCTTCCAAACCTTTGAC-3ʹ
IL-1β reverse	5ʹ-AGGCCACAGGTATTTTGTCG-3ʹ
TNFα forward	5ʹ-CCACCACGCTCTTCTGTCTA-3ʹ
TNFα reverse	5ʹ-CACTTGGTGGTTTGCTACGA-3ʹ
IL-6 forward	5ʹ-TCCAGTTGCCTTCTTGGGAC-3ʹ
IL-6 reverse	5ʹ-GTACTCCAGAAGACCAGAGG-3ʹ
GAPDH forward	5ʹ-ACCACAGTCCATGCCATCAC-3ʹ
GAPDH reverse	5ʹ-TCCACCACCCTGTTGCTGTA-3ʹ

### Statistical analysis

All data are presented as mean ± SEM. Each experiment was performed in triplicate, where appropriate. The data were analyzed by either the unpaired Student’s t-test or one-way analysis of variance, which was followed by the Tukey honestly significant difference multiple-comparison post hoc test. When the Levene test for unequal variance was significant, a post hoc Dunnett T3 was used. All statistical analyses were performed using GraphPad Prism 6.0 (GraphPad Software, Inc, Boston, MA, USA). A value of p < 0.05 was considered statistically significant.

## Conclusion

Hearing thresholds were gradually increased in *Col4a3* KO mice, while the TSA, a HDACi, ameliorated this hearing impairment in AS mice. In AS mice, oxidative stress markers 4-HNE and 3-NT were increased, while TSA treatment effectively reduced them. Furthermore, upon induction of inflammation by siCol4α3 transfection, TSA treatment reduced inflammatory cytokine levels. Additionally, TSA mitigated the thickening of the stria vascularis basement membrane induced by *Col4a3* KO. Thus, TSA is a potential therapeutic agent for mitigating hearing deterioration in an AS model.

## Supporting information

S1 Fig Systemic injection of TSA shows no significant changes in body weight. Trichostatin A (TSA, 10 mg/kg body weight, once daily) treatment was administered by intraperitoneal (IP) injection to wild-type (WT) and *Col4a3* KO (KO) mice from 3 weeks to 7 weeks (28 days). There was no significant change in body weight for both WT and *Col4a3* KO mice, regardless of whether they were in the control or TSA-treated group (mouse number WT-CON = 13, WT-TSA = 6, KO-CON = 9 and KO-TSA = 17). Data are the mean ± S.E.M (standard error of the mean).
(JPG)

S2 Fig Cell viability assessment by MTT assay. Trichostatin A (TSA) does not induce IC50 toxicity at under 50μM treatment in HEK293T cells. Data are the mean ± S.E.M. (n = 3).
(JPG)

S1 Raw images (ZIP)

## List of abbreviations

4-HNE: 4-Hydroxynonenal
3-NT: 3-Nitrotyrosine
ABR: Auditory Brainstem Response
AS: Alport Syndrome
ESRD: End Stage Renal Disease
GHL: Genetic Hearing Loss
HDACi: Histone Deacetylase Inhibitor
HEI-OC1 cell: House Ear Institute-Organ of Corti 1 cell
HEK293T cell: Human Embryonic Kidney 293 cell containing T antigen
HO: Heme Oxygenase
Keap1: Kelch-like ECH-associated protein 1
KO: Knock Out
NGS: Next Generation Sequencing
NRF 2: Nuclear factor erythroid 2-related factor 2
SAHA: Suberoylanilide hydroxamic acid
siRNA: small interfering RNA
SV: Stria Vascularis
TEM: Transmission Electron Microscopy
TSA: Trichostatin A
WT: Wild Type
