# Supplementary figures and images for: Trichostatin A suppresses hearing loss by reducing oxidative stress and inflammation in an Alport syndrome model
Source: PLoS One. 2025 Feb 5;20(2):e0316033. doi: 10.1371/journal.pone.0316033 (PMC11798502; doi:10.1371/journal.pone.0316033)

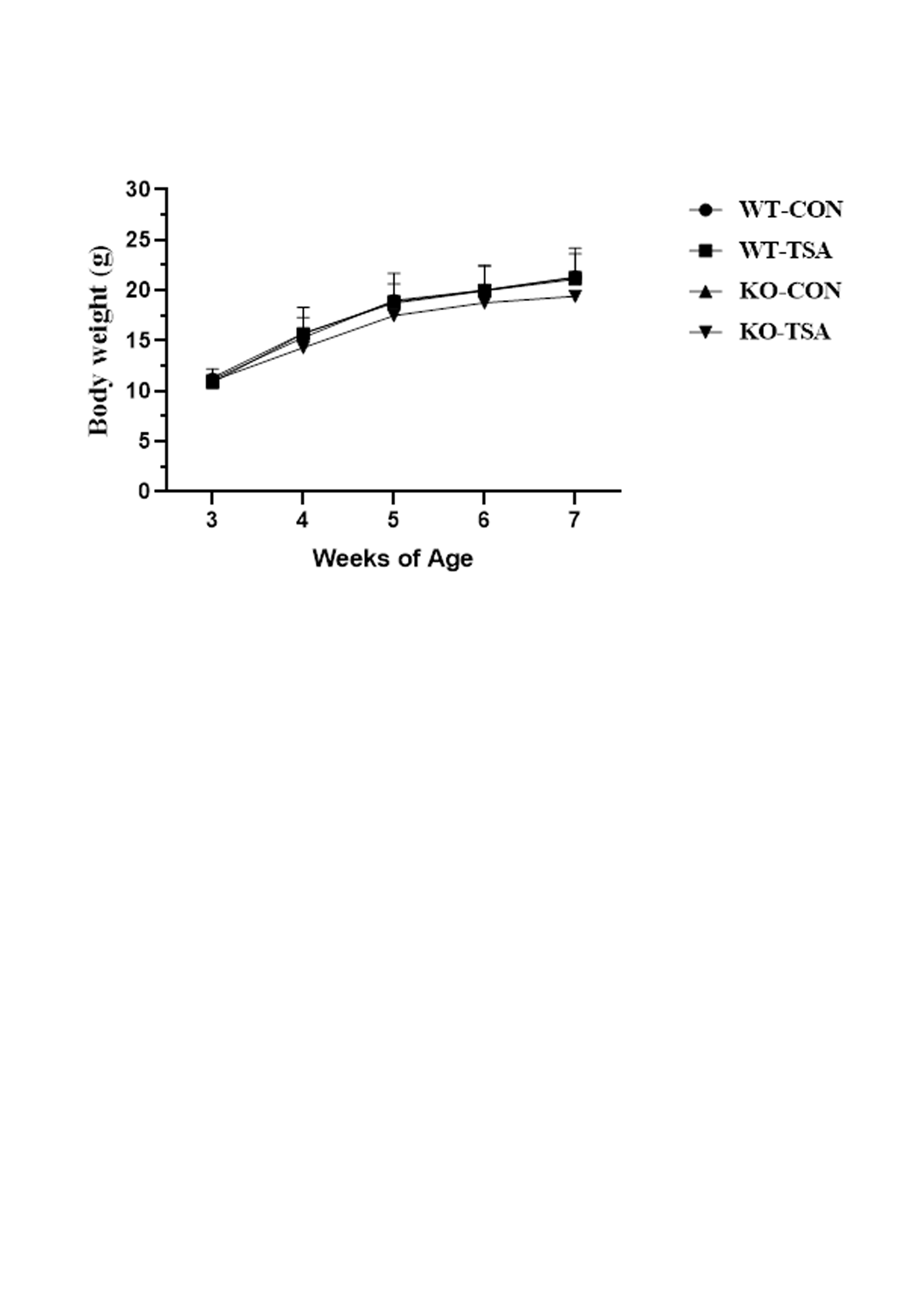

Supplement: S1 Fig — Trichostatin A (TSA, 10 mg/kg body weight, once daily) treatment was administered by intraperitoneal (IP) injection to wild-type (WT) and Col4a3 KO (KO) mice from 3 weeks to 7 weeks (28 days). There was no significant change in body weight for both WT and Col4a3 KO mice, regardless of whether they were in the control or TSA-treated group (mouse number WT-CON = 13, WT-TSA = 6, KO-CON = 9 and KO-TSA = 17). Data are the mean ± S.E.M (standard error of the mean). (JPG) [file pone.0316033.s001.jpg]

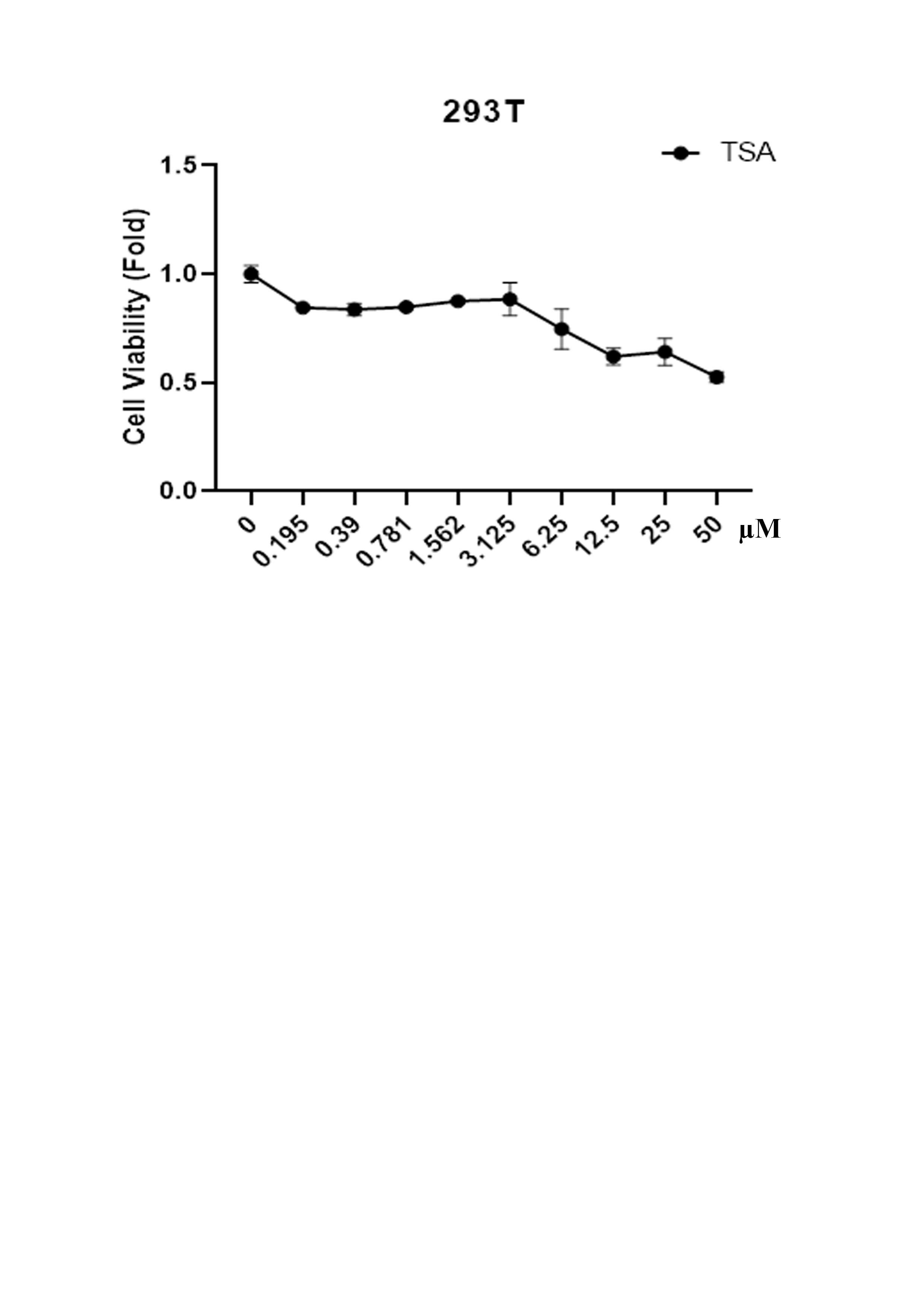

Supplement: S2 Fig — Trichostatin A (TSA) does not induce IC50 toxicity at under 50μM treatment in HEK293T cells. Data are the mean ± S.E.M. (n = 3). (JPG) [file pone.0316033.s002.jpg]

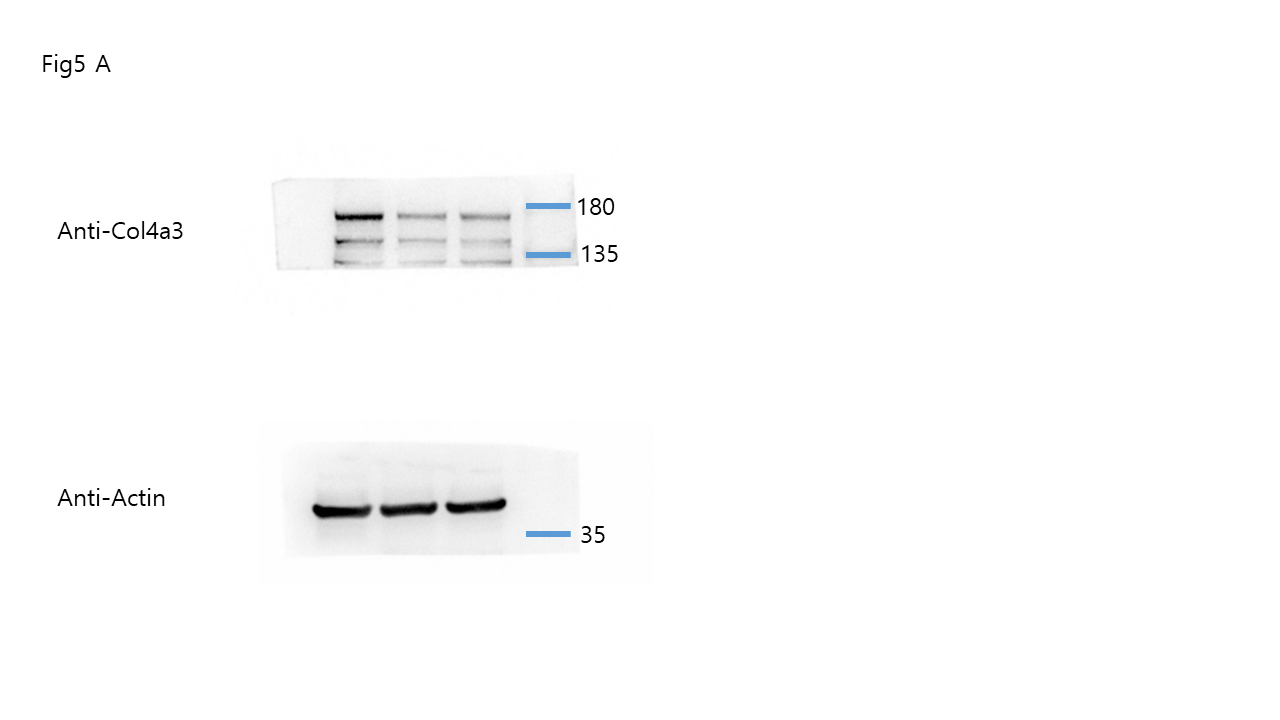

Supplement: S1 Raw images — (ZIP) [file pone.0316033.s003.zip › WB full blot band marker check v2/Fig 5A.PNG]

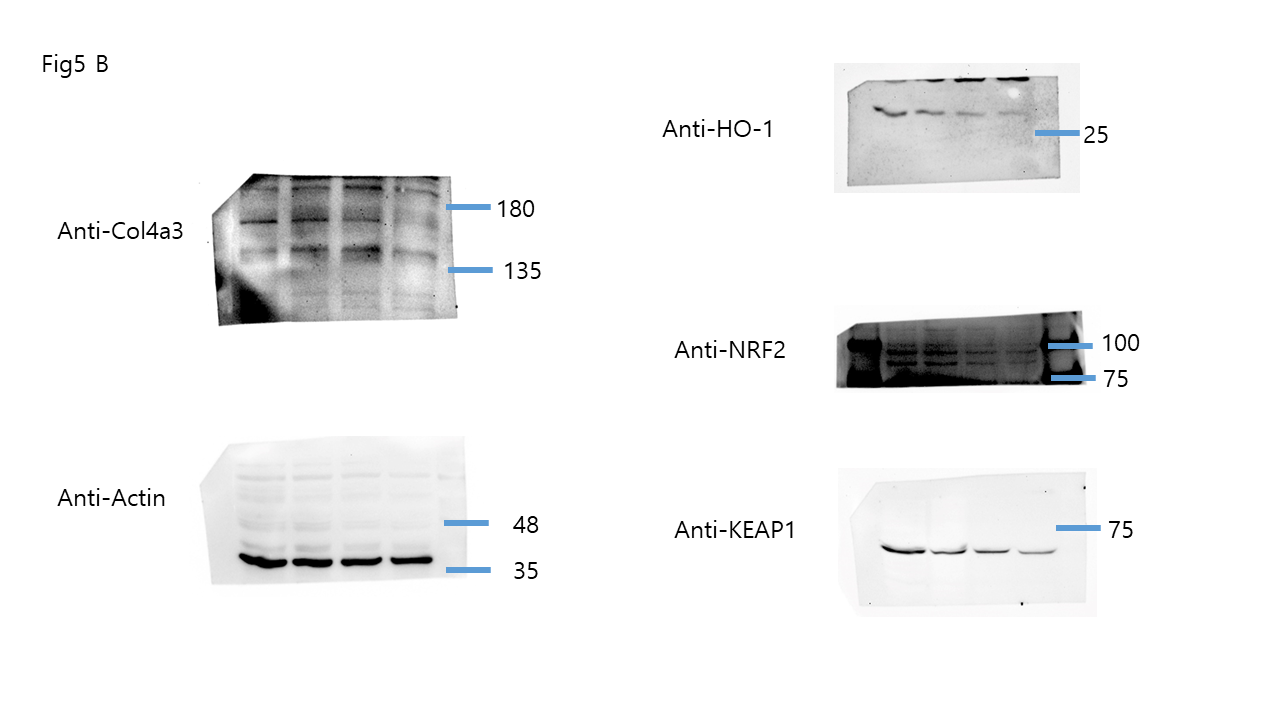

Supplement: S1 Raw images — (ZIP) [file pone.0316033.s003.zip › WB full blot band marker check v2/Fig 5B.PNG]

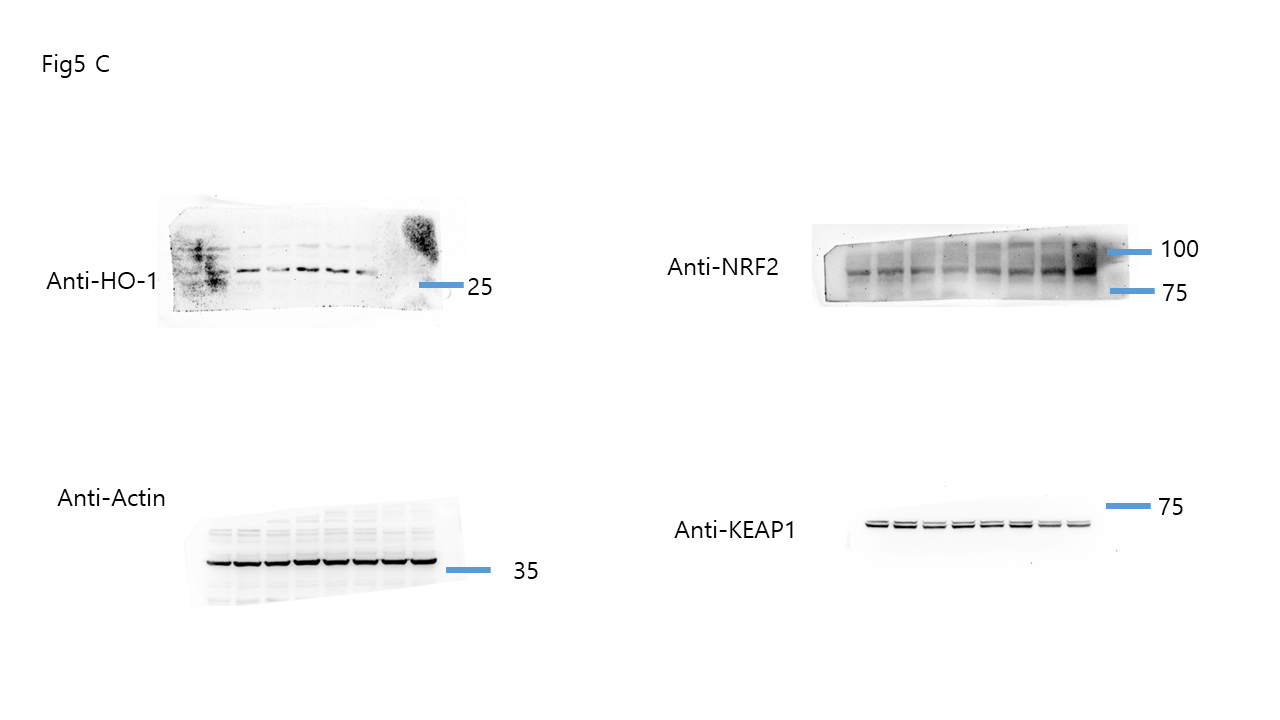

Supplement: S1 Raw images — (ZIP) [file pone.0316033.s003.zip › WB full blot band marker check v2/Fig 5C.PNG]

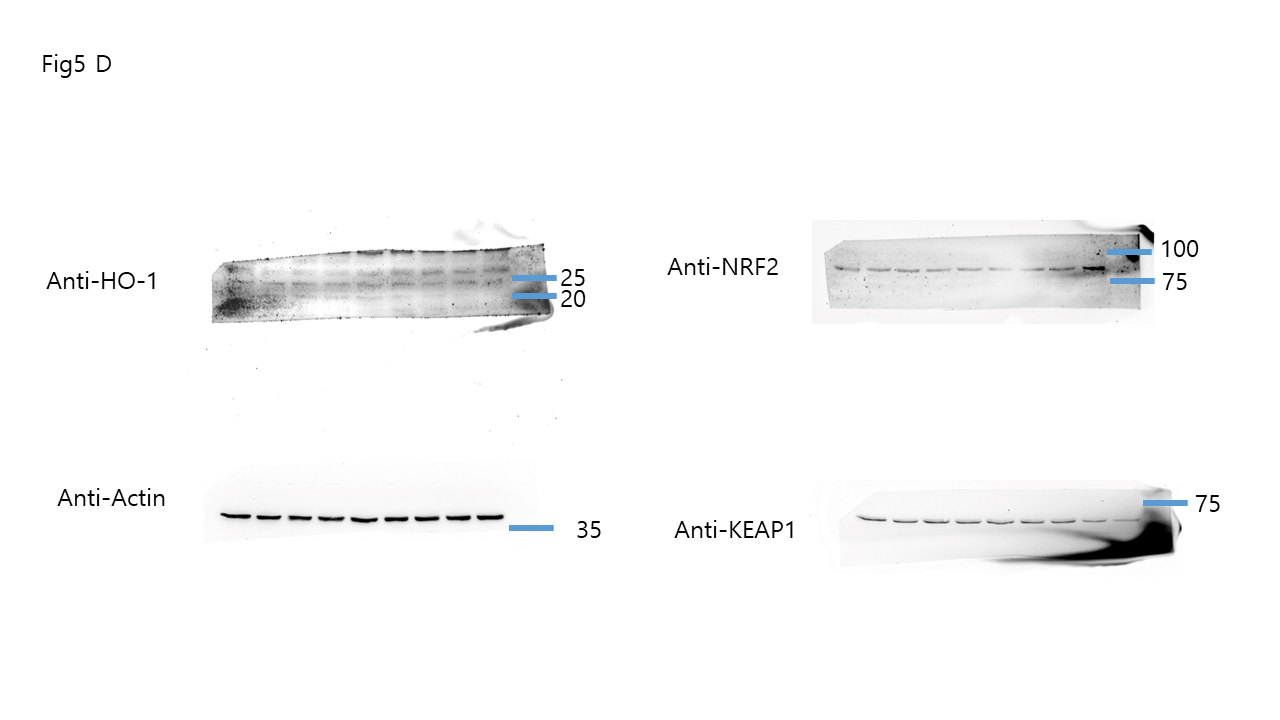

Supplement: S1 Raw images — (ZIP) [file pone.0316033.s003.zip › WB full blot band marker check v2/Fig 5D.PNG]

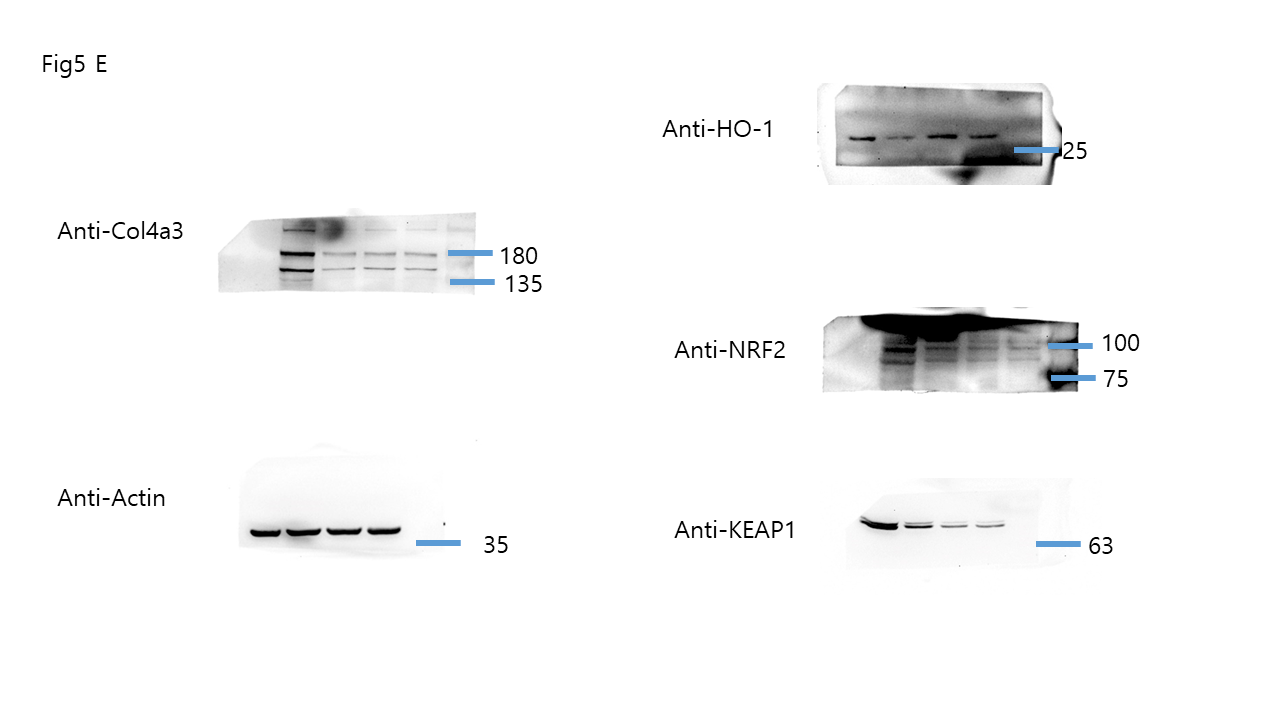

Supplement: S1 Raw images — (ZIP) [file pone.0316033.s003.zip › WB full blot band marker check v2/Fig 5E.PNG]

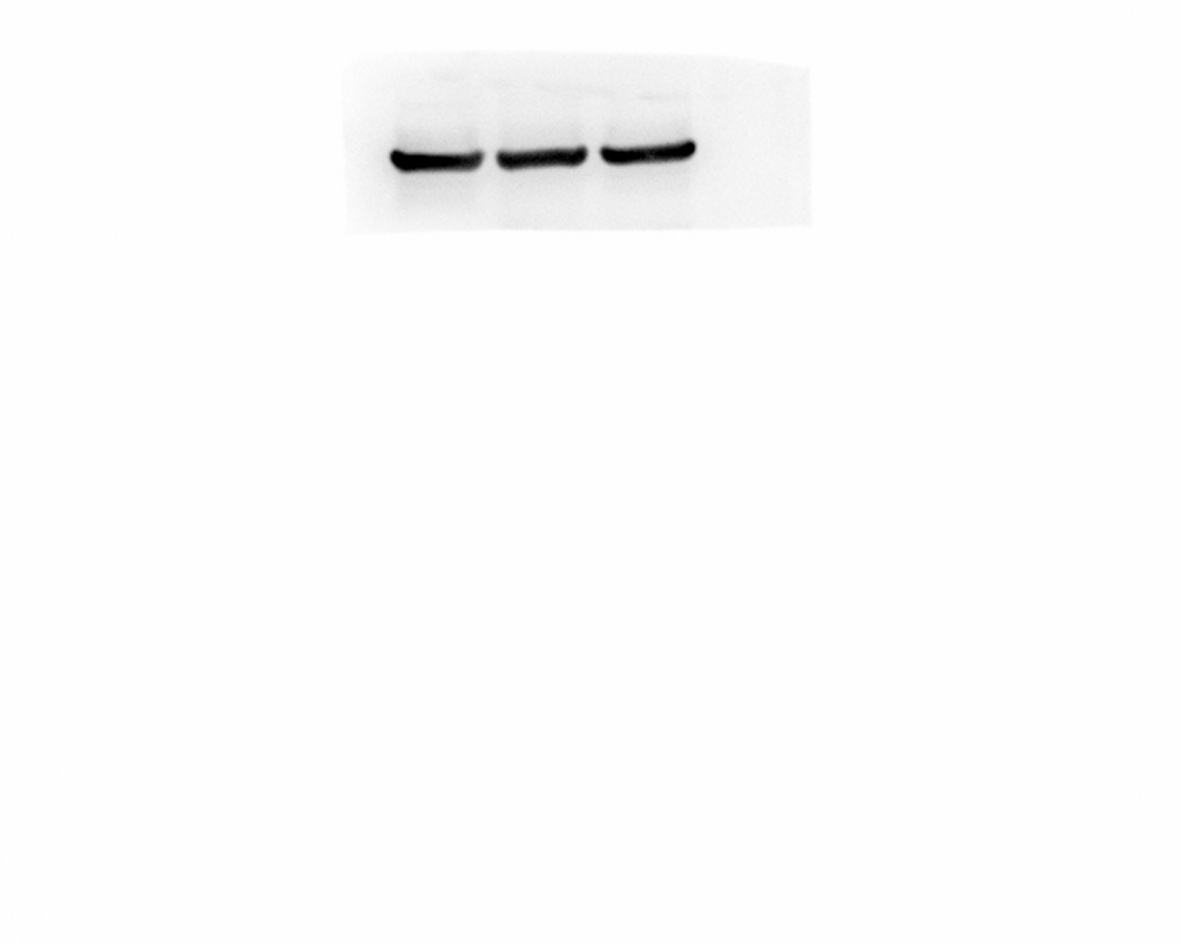

Supplement: S1 Raw images — (ZIP) [file pone.0316033.s003.zip › WB full blot band marker check v2/Figure5 A - Actin.jpg]

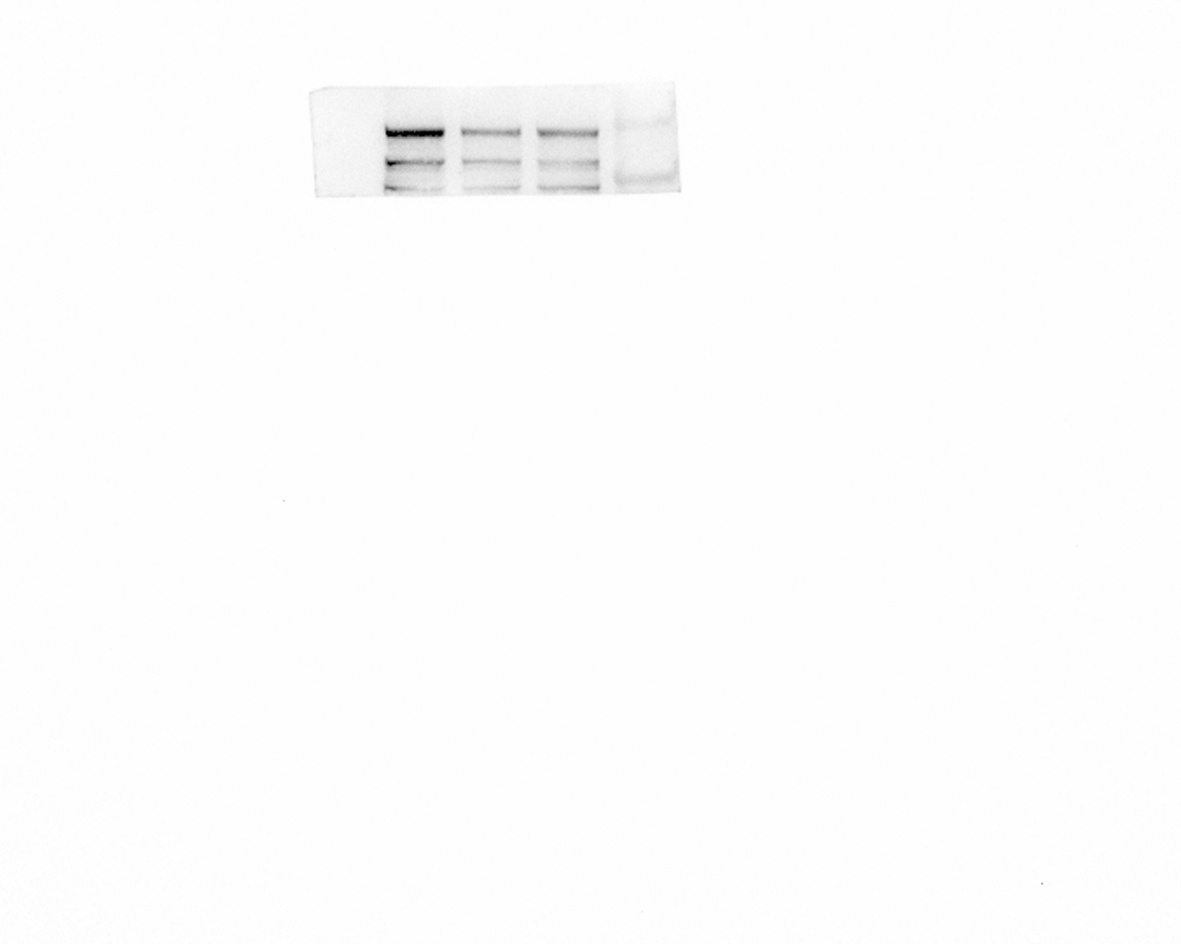

Supplement: S1 Raw images — (ZIP) [file pone.0316033.s003.zip › WB full blot band marker check v2/Figure5 A - Col4a3.jpg]

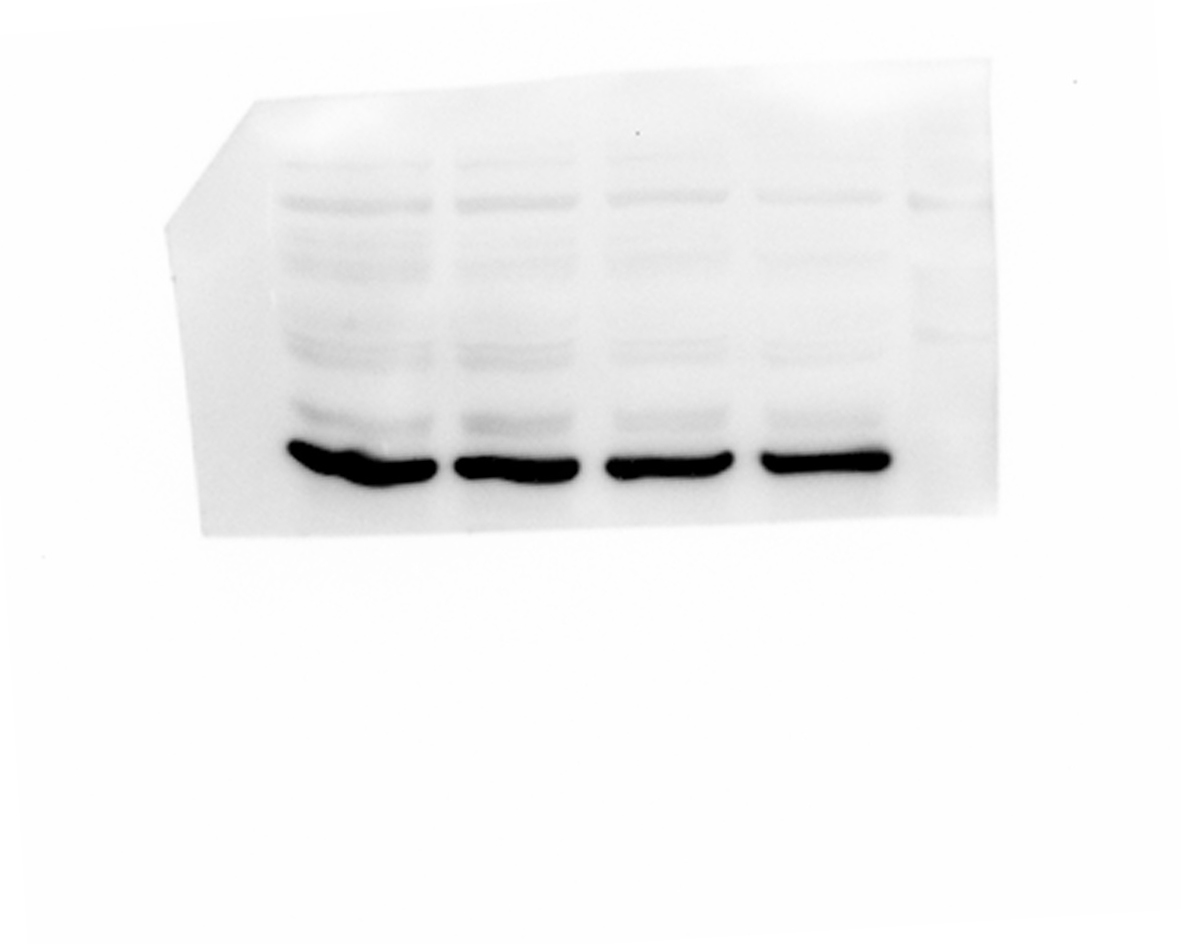

Supplement: S1 Raw images — (ZIP) [file pone.0316033.s003.zip › WB full blot band marker check v2/Figure5 B - Actin.jpg]

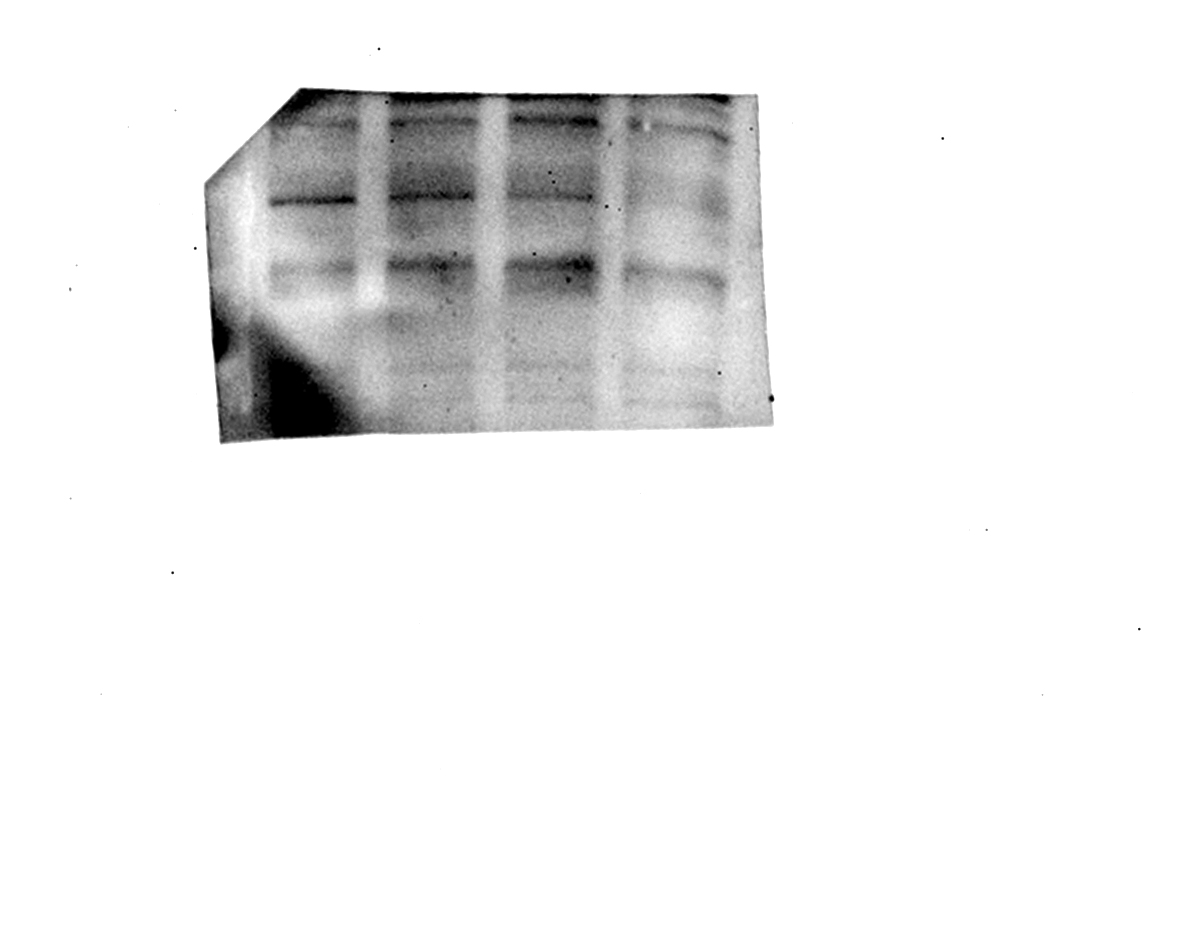

Supplement: S1 Raw images — (ZIP) [file pone.0316033.s003.zip › WB full blot band marker check v2/Figure5 B - Col4a3.jpg]

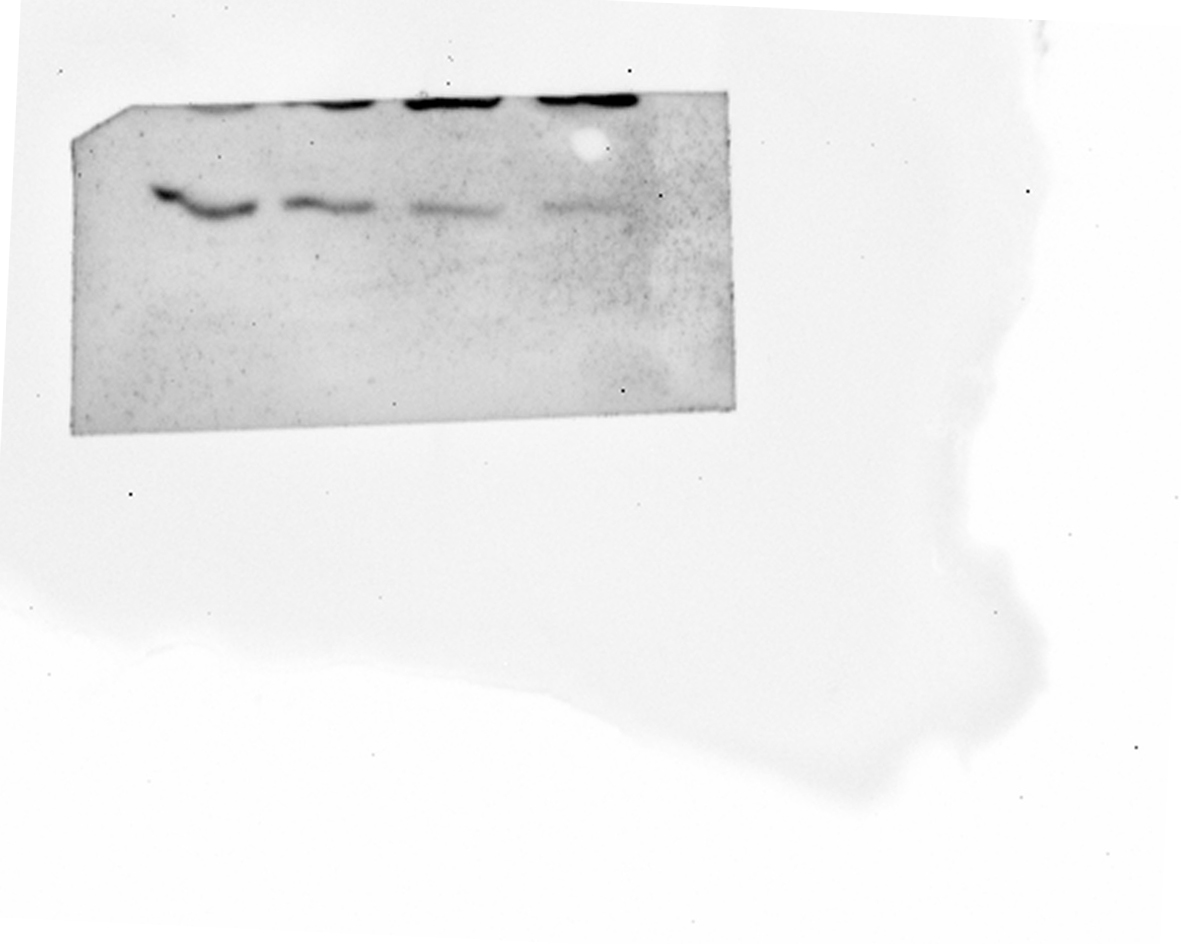

Supplement: S1 Raw images — (ZIP) [file pone.0316033.s003.zip › WB full blot band marker check v2/Figure5 B - HO-1.jpg]

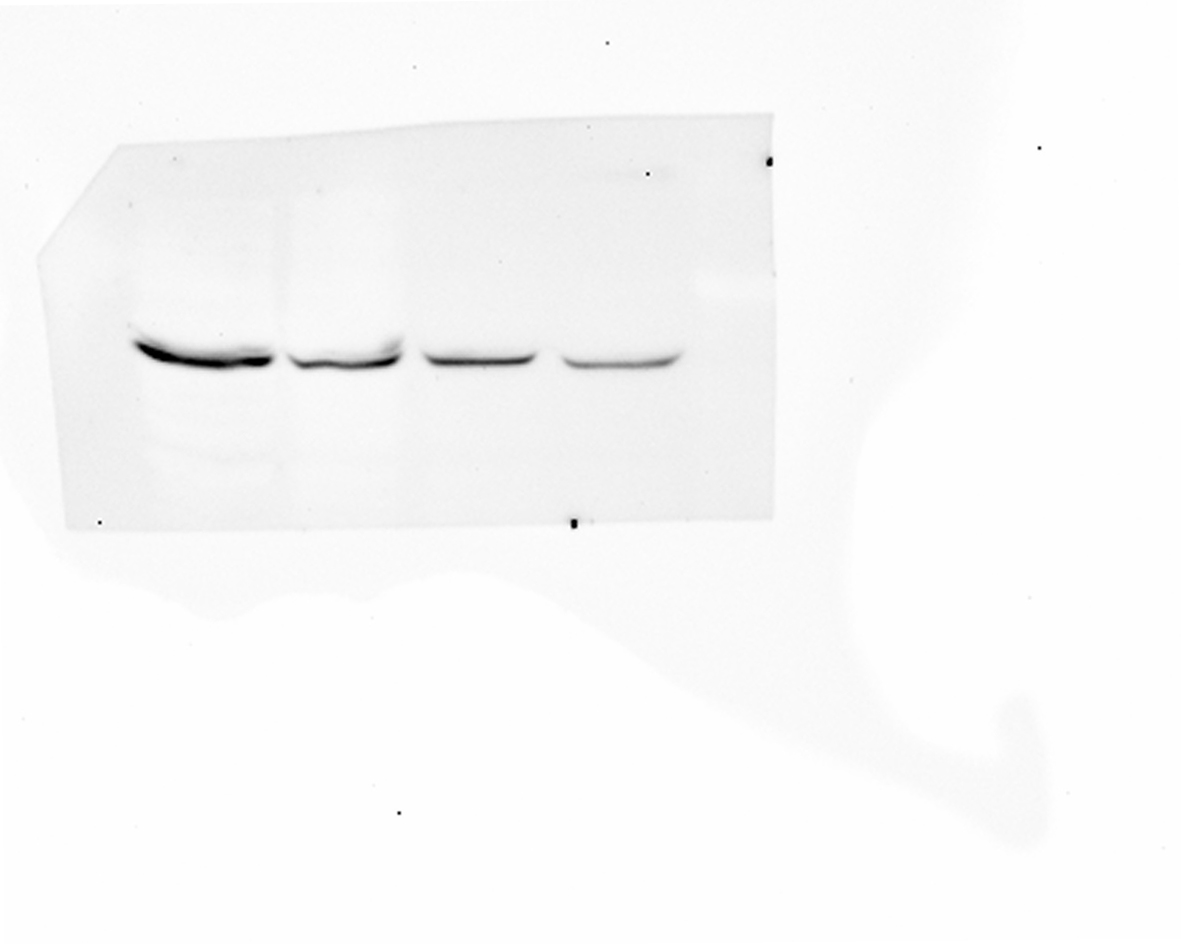

Supplement: S1 Raw images — (ZIP) [file pone.0316033.s003.zip › WB full blot band marker check v2/Figure5 B - KEAP1.jpg]

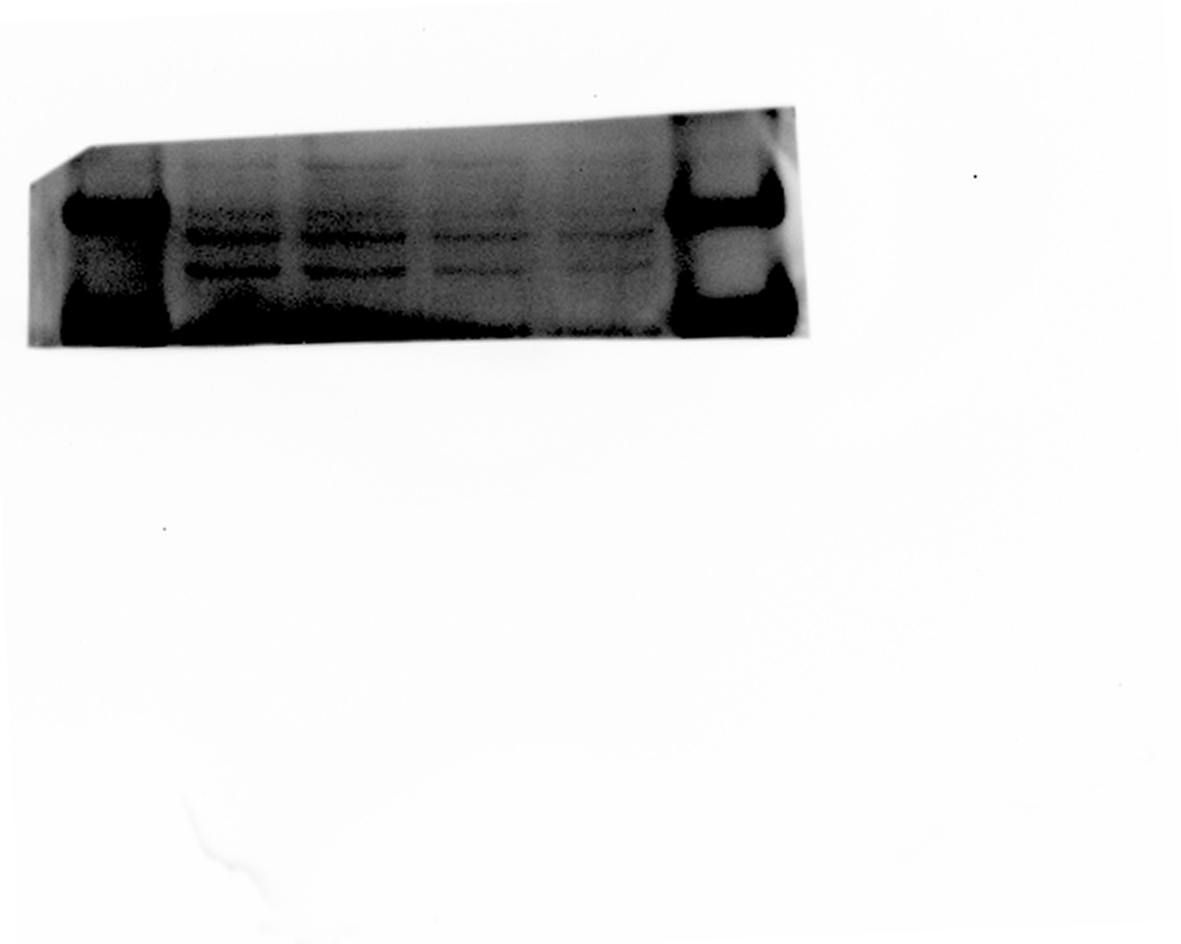

Supplement: S1 Raw images — (ZIP) [file pone.0316033.s003.zip › WB full blot band marker check v2/Figure5 B - NRF2.jpg]

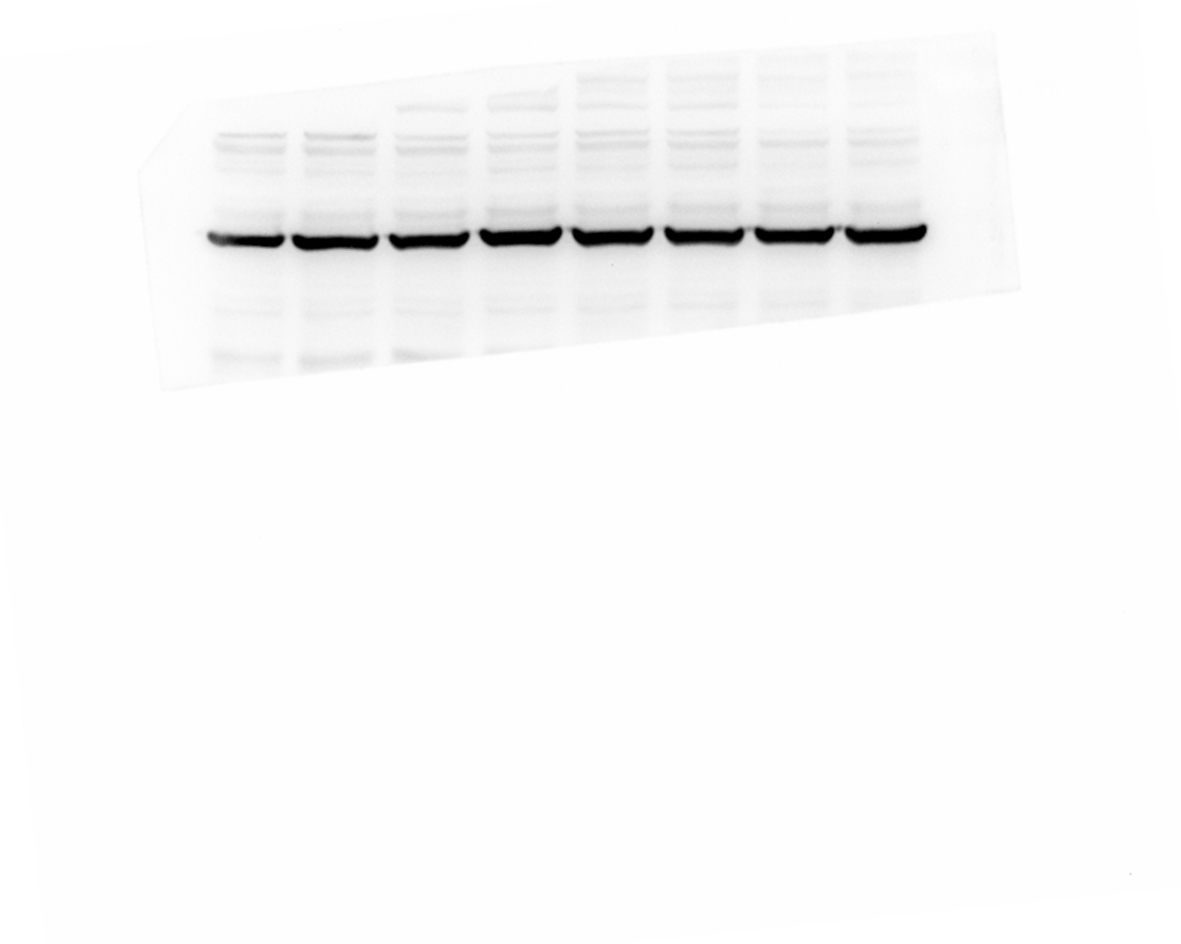

Supplement: S1 Raw images — (ZIP) [file pone.0316033.s003.zip › WB full blot band marker check v2/Figure5 C - Actin.jpg]

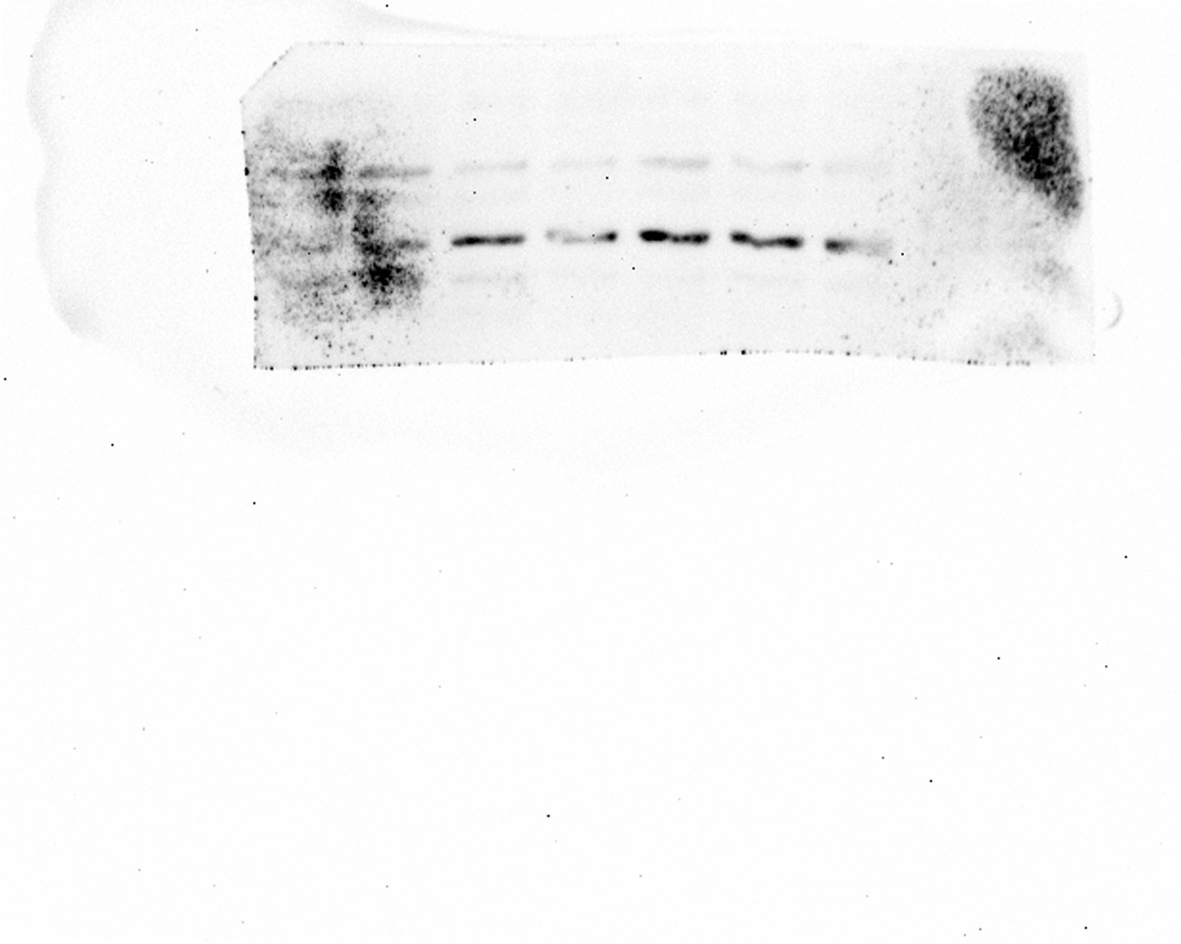

Supplement: S1 Raw images — (ZIP) [file pone.0316033.s003.zip › WB full blot band marker check v2/Figure5 C - HO-1.jpg]

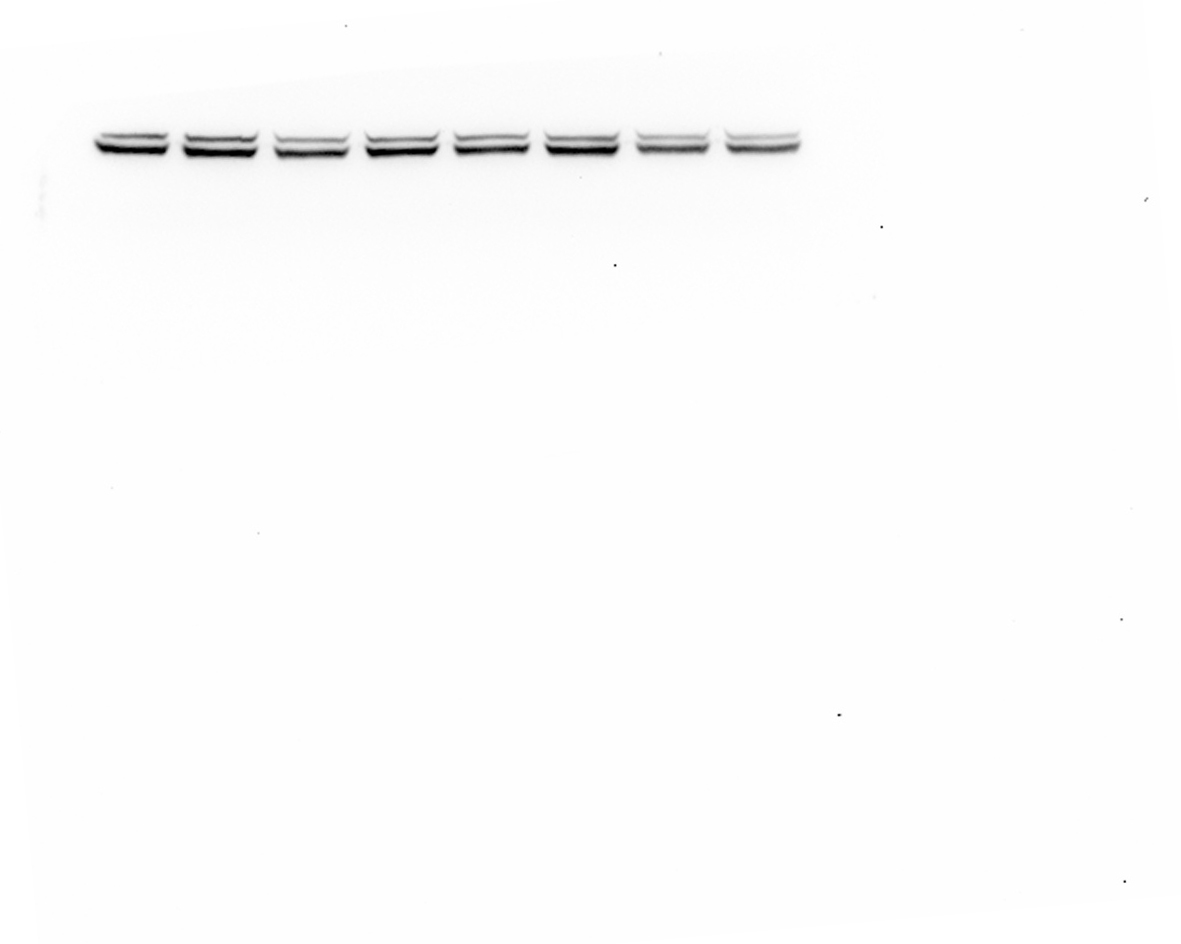

Supplement: S1 Raw images — (ZIP) [file pone.0316033.s003.zip › WB full blot band marker check v2/Figure5 C - KEAP1.jpg]

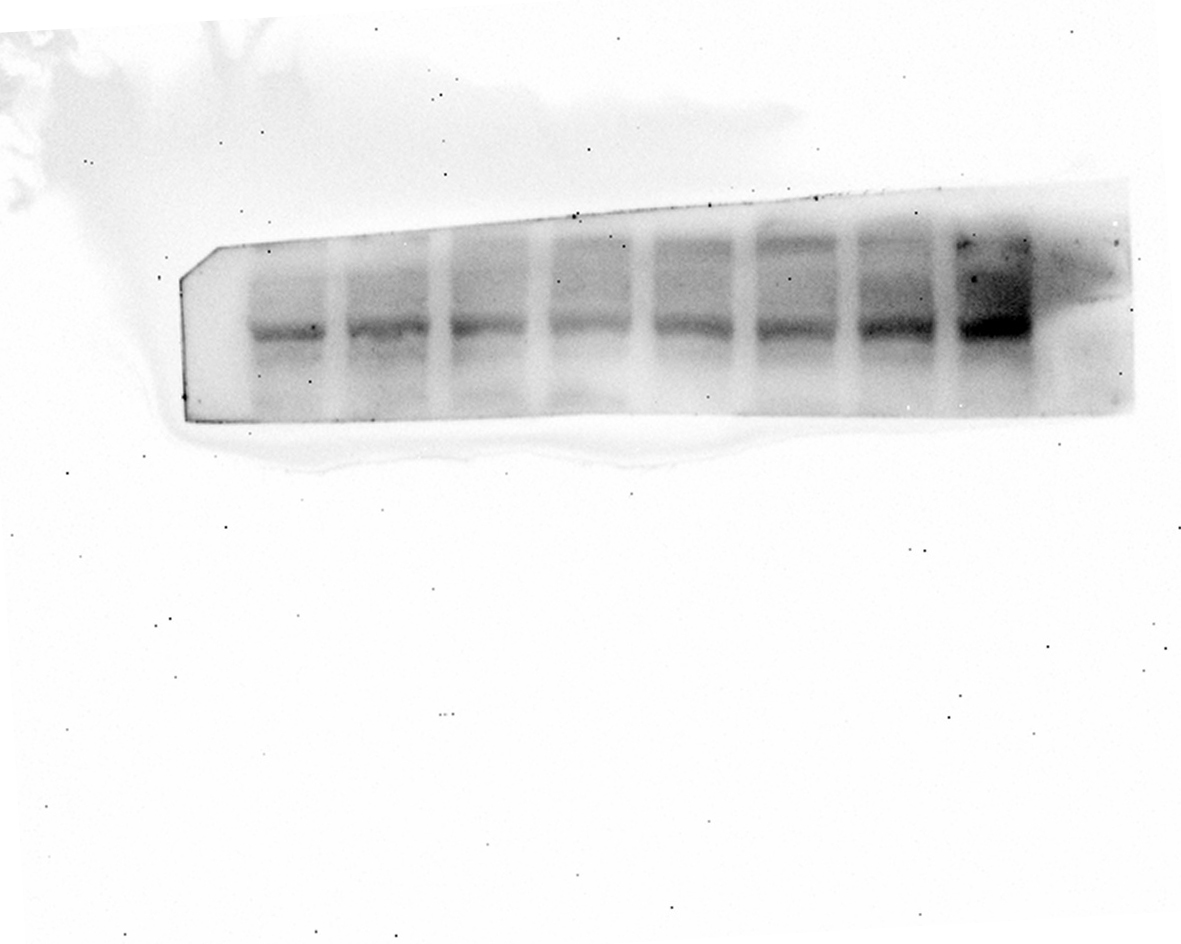

Supplement: S1 Raw images — (ZIP) [file pone.0316033.s003.zip › WB full blot band marker check v2/Figure5 C - NRF2.jpg]

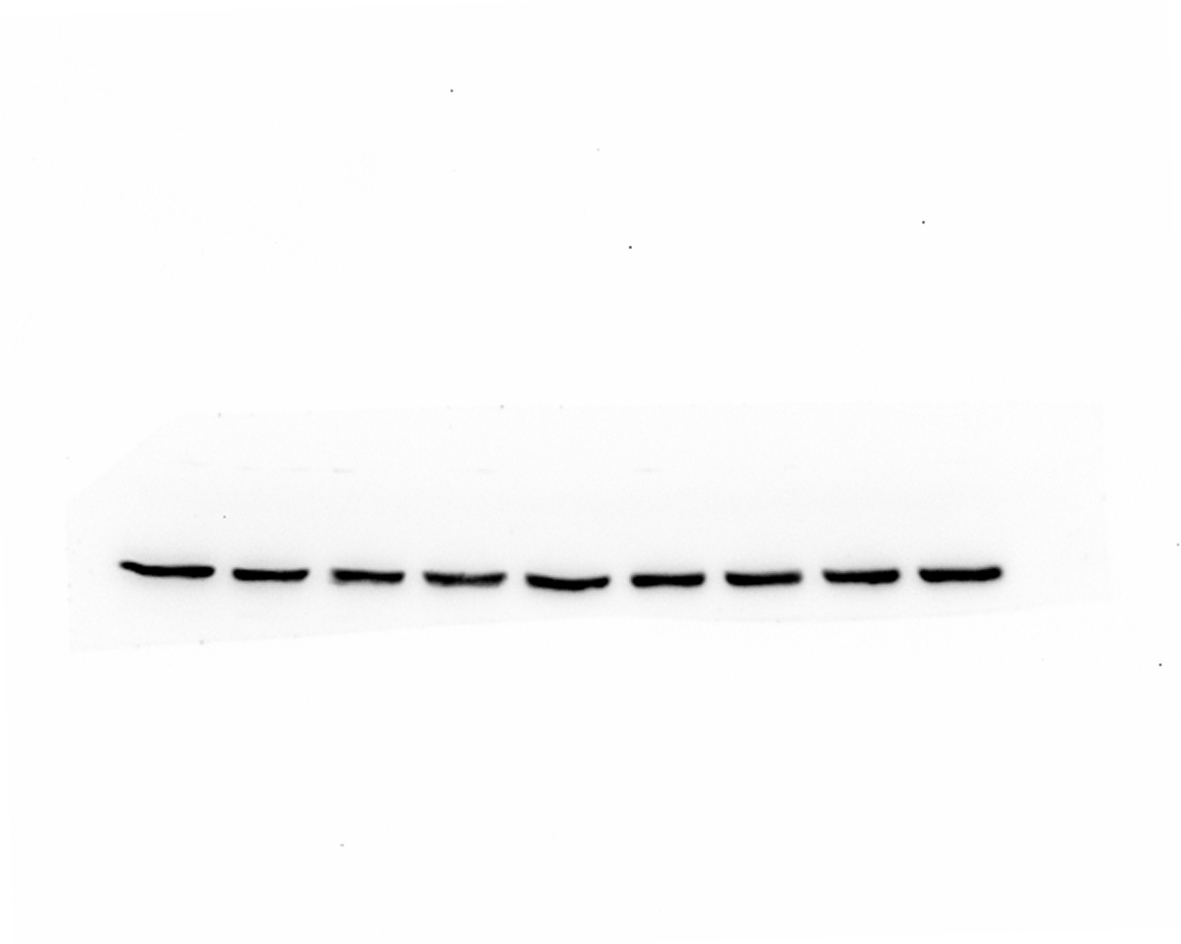

Supplement: S1 Raw images — (ZIP) [file pone.0316033.s003.zip › WB full blot band marker check v2/Figure5 D - Actin.jpg]

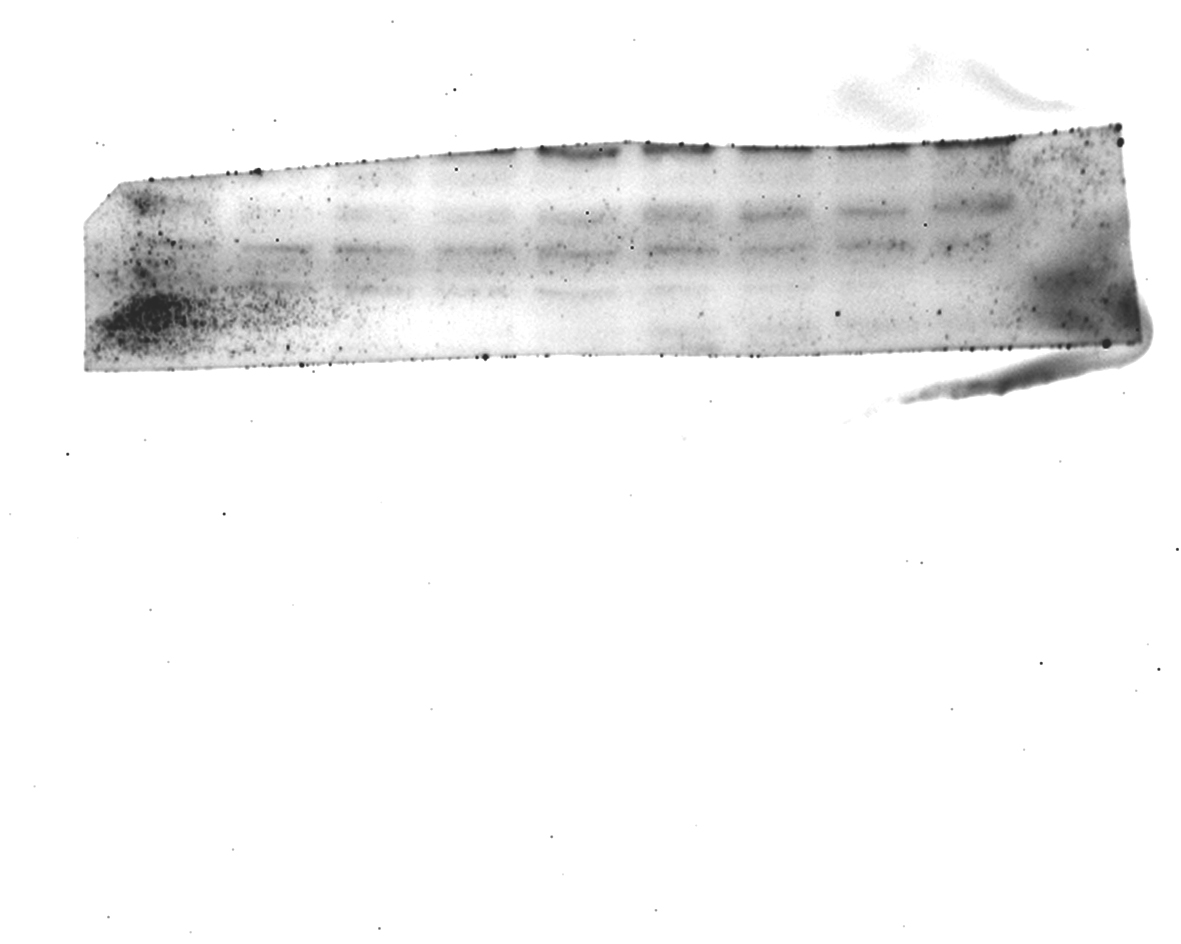

Supplement: S1 Raw images — (ZIP) [file pone.0316033.s003.zip › WB full blot band marker check v2/Figure5 D - HO-1.jpg]

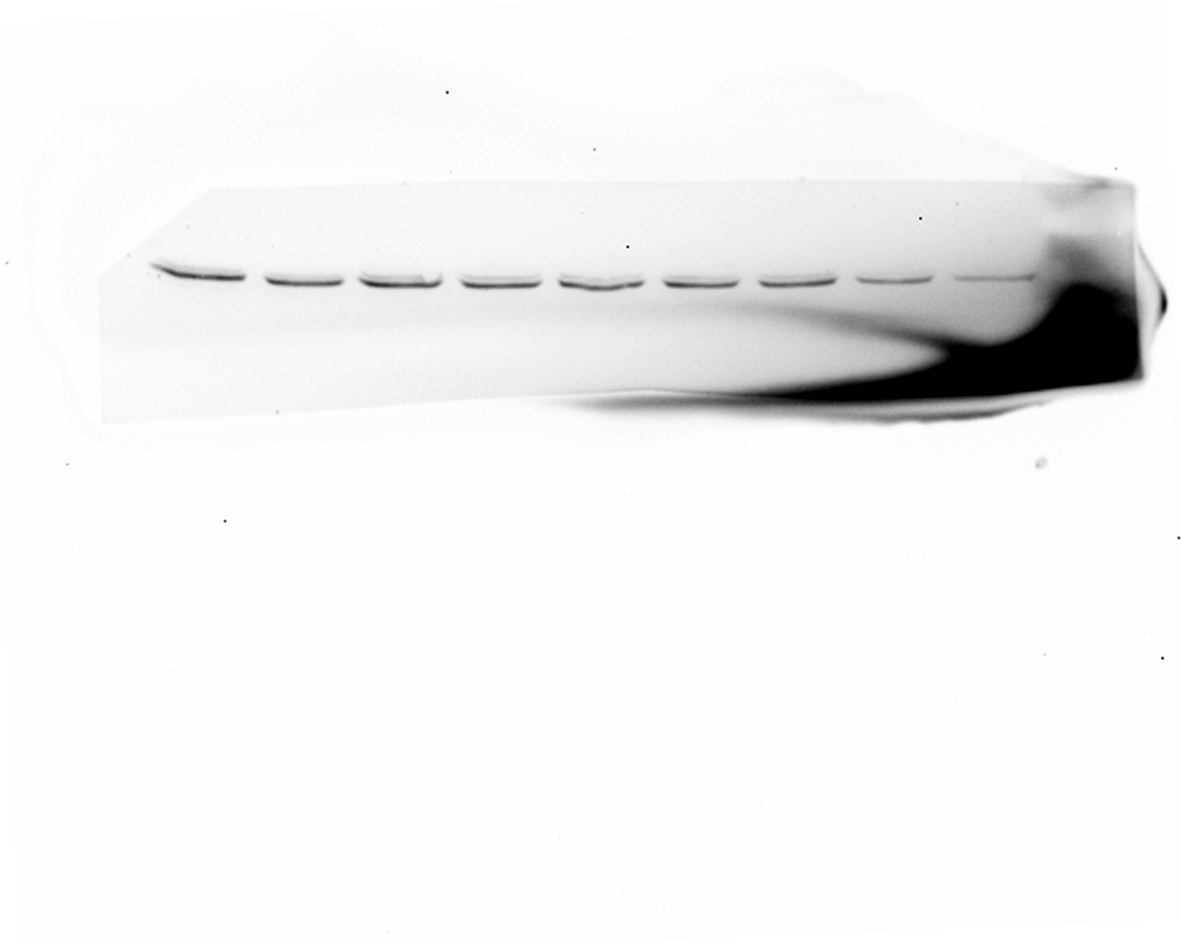

Supplement: S1 Raw images — (ZIP) [file pone.0316033.s003.zip › WB full blot band marker check v2/Figure5 D - KEAP1.jpg]

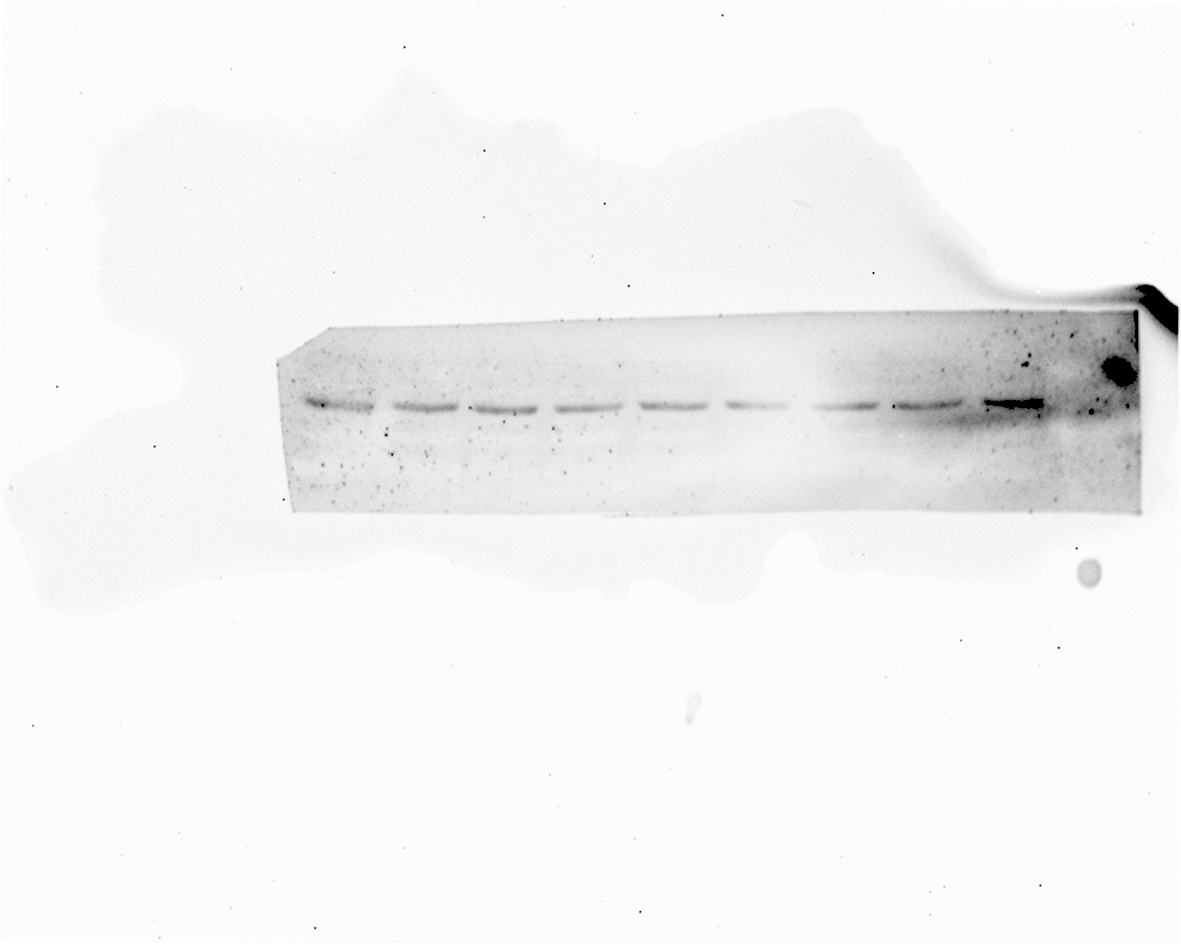

Supplement: S1 Raw images — (ZIP) [file pone.0316033.s003.zip › WB full blot band marker check v2/Figure5 D - NRF2.jpg]

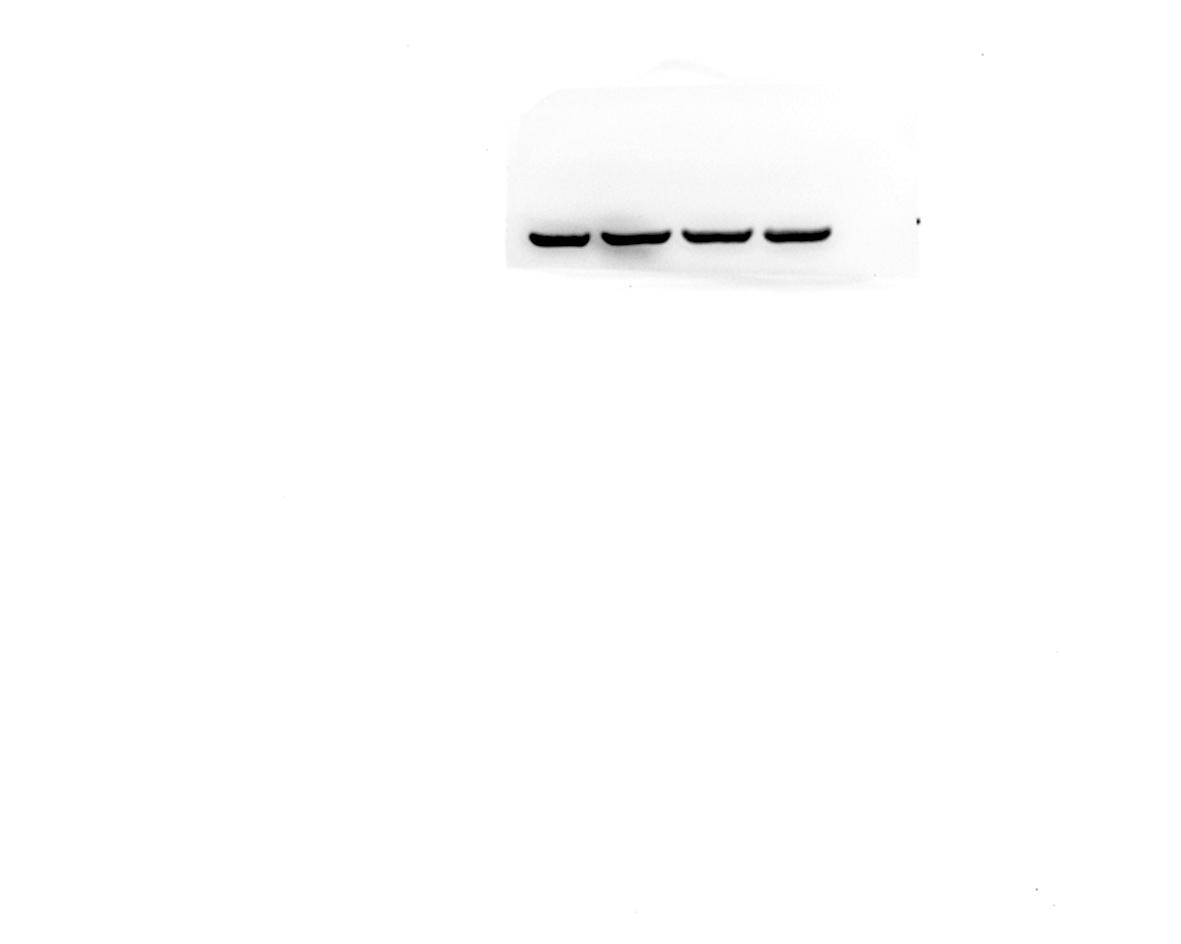

Supplement: S1 Raw images — (ZIP) [file pone.0316033.s003.zip › WB full blot band marker check v2/Figure5 E - Actin.jpg]

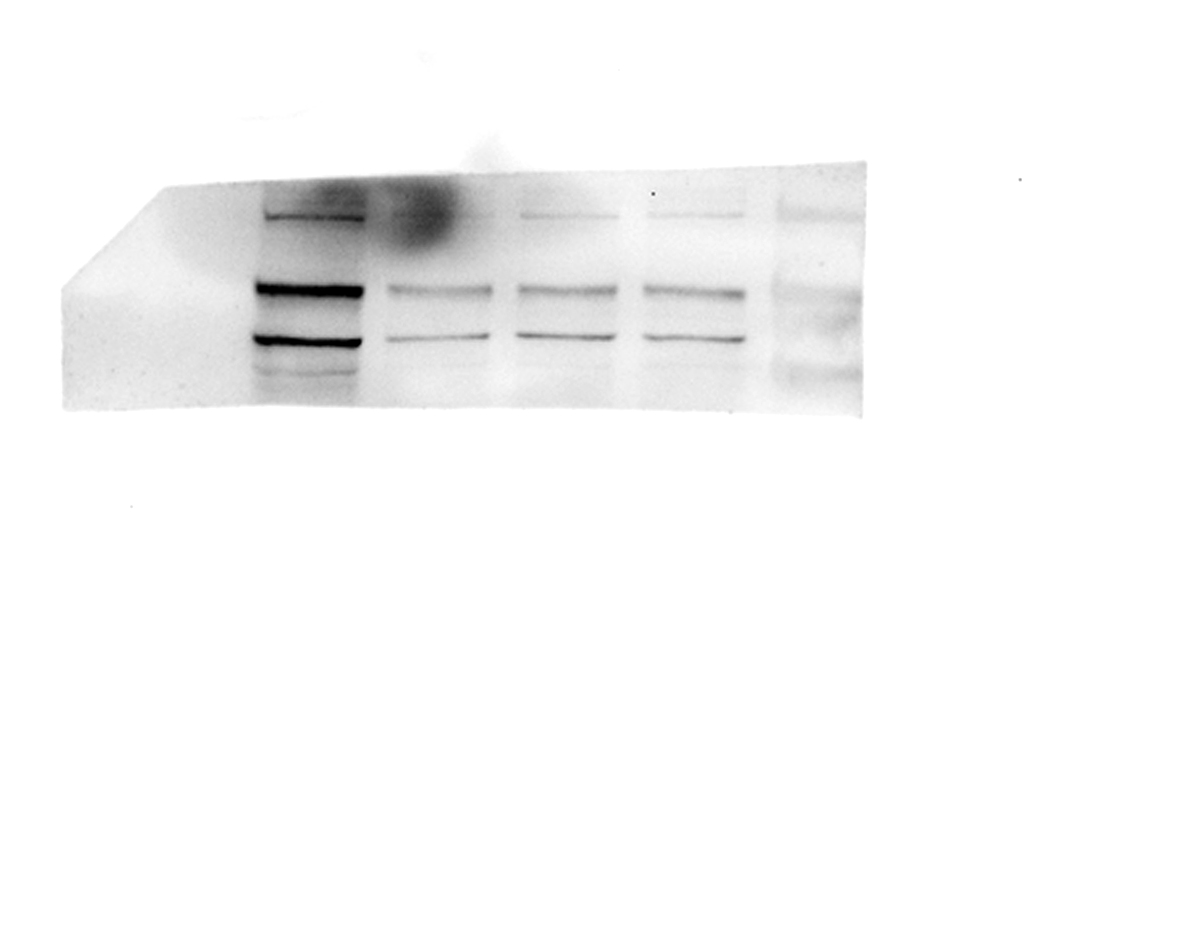

Supplement: S1 Raw images — (ZIP) [file pone.0316033.s003.zip › WB full blot band marker check v2/Figure5 E - Col4a3.jpg]

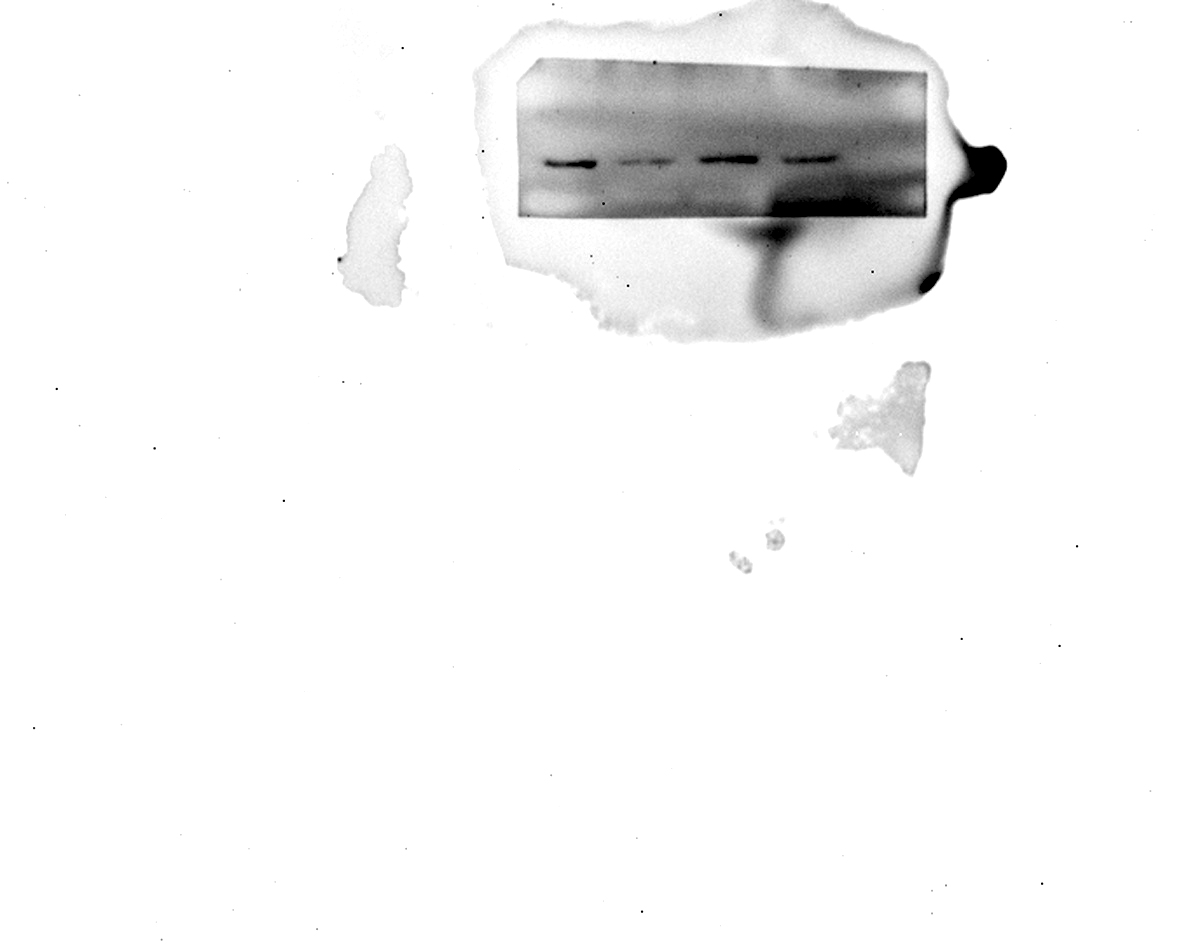

Supplement: S1 Raw images — (ZIP) [file pone.0316033.s003.zip › WB full blot band marker check v2/Figure5 E - HO-1.jpg]

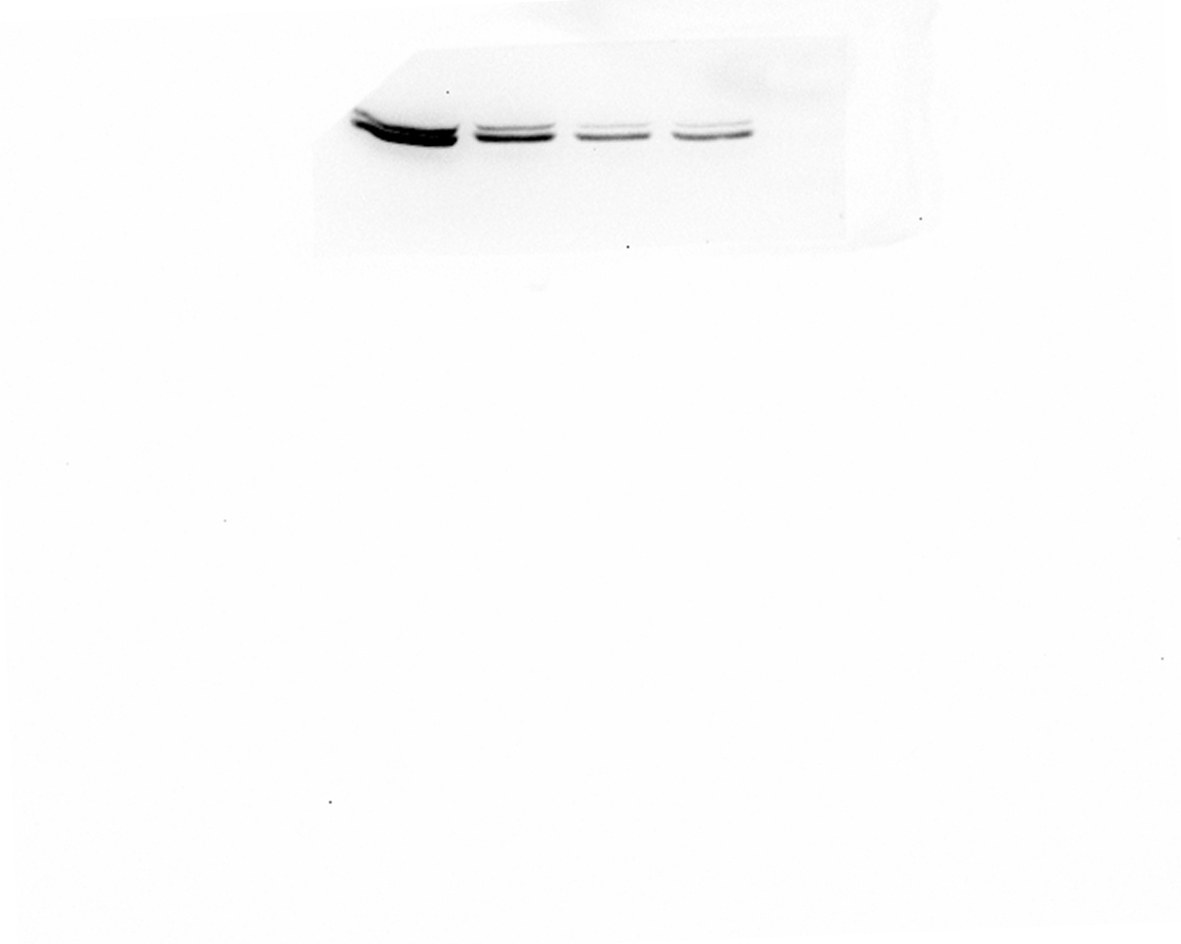

Supplement: S1 Raw images — (ZIP) [file pone.0316033.s003.zip › WB full blot band marker check v2/Figure5 E - KEAP1.jpg]

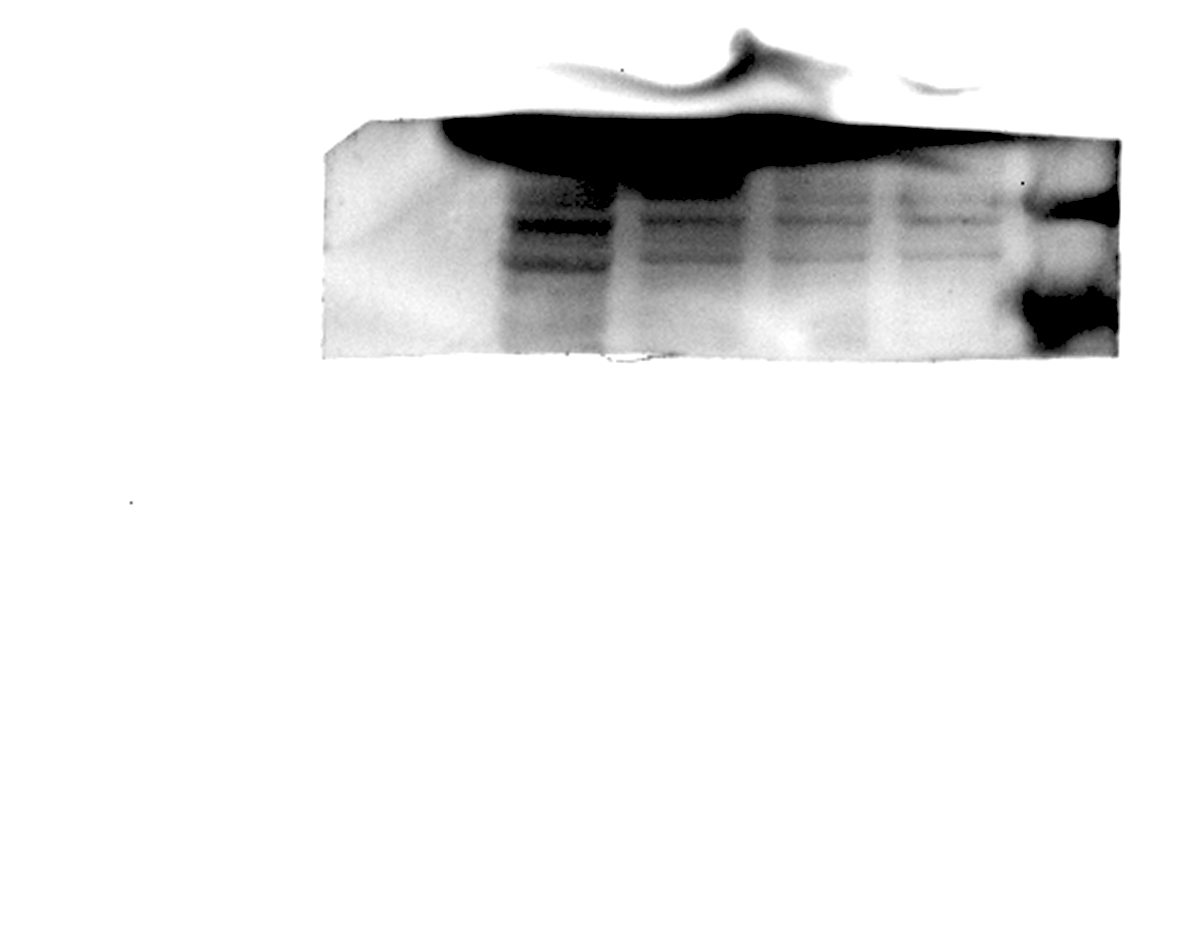

Supplement: S1 Raw images — (ZIP) [file pone.0316033.s003.zip › WB full blot band marker check v2/Figure5 E - NRF2.jpg]

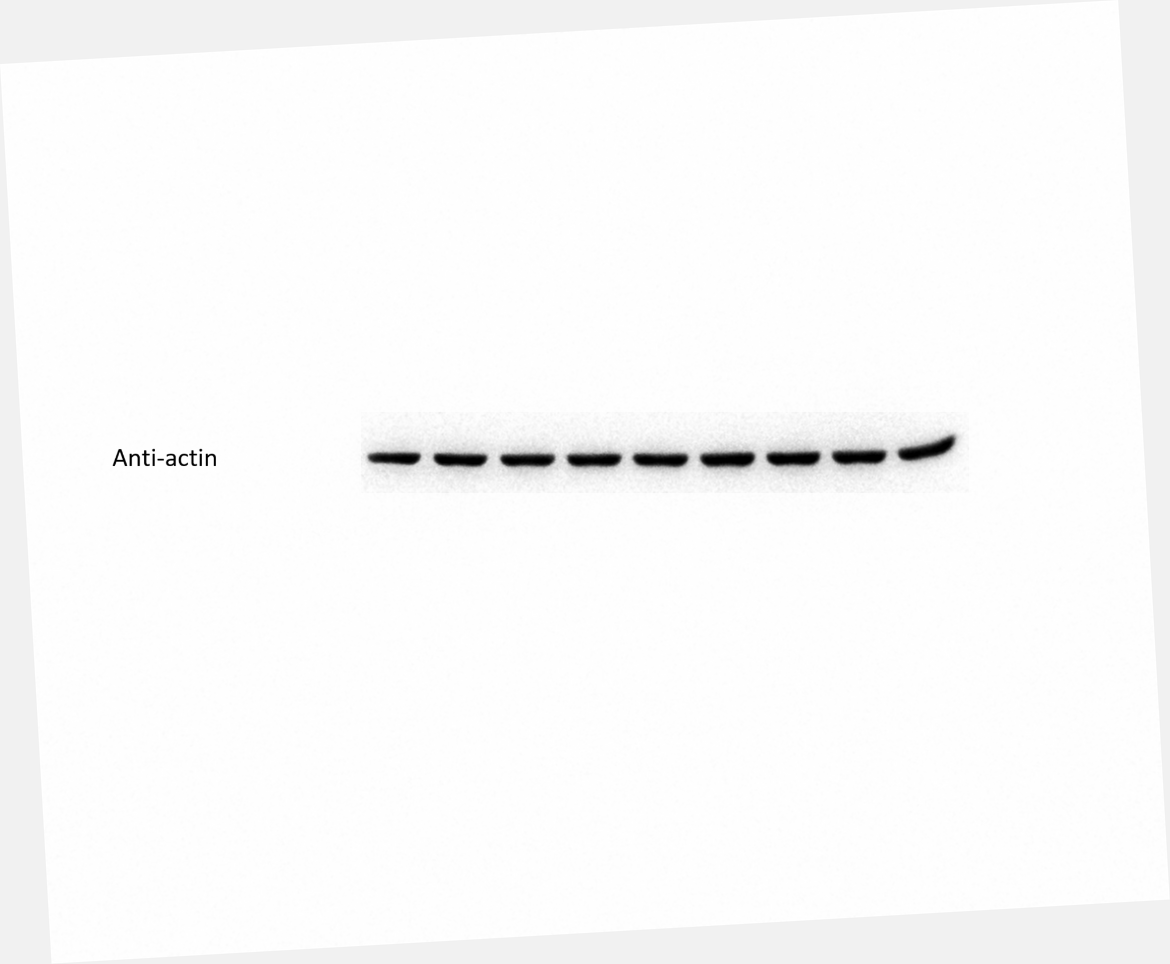

Supplement: S1 Raw images — (ZIP) [file pone.0316033.s003.zip › WB full blot band marker check v2/Figure6 actin.tif]

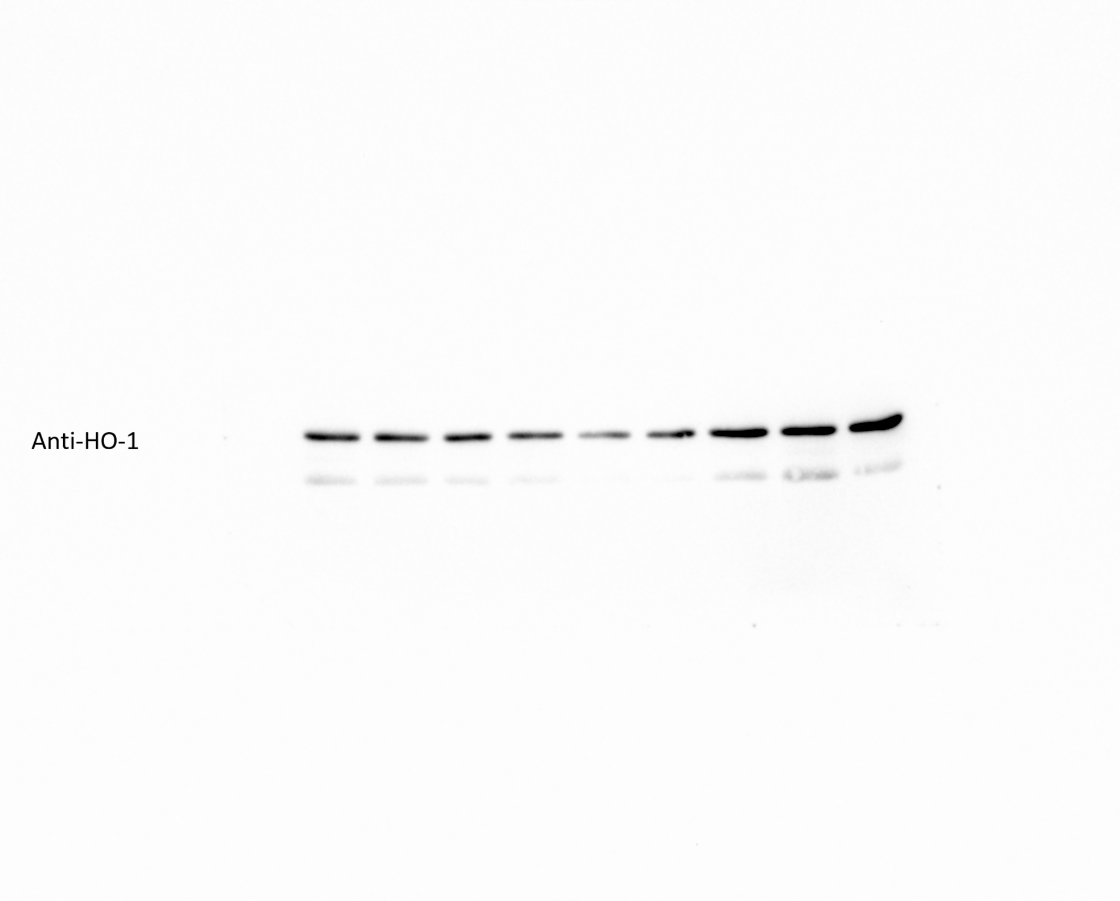

Supplement: S1 Raw images — (ZIP) [file pone.0316033.s003.zip › WB full blot band marker check v2/Figure6 HO-1.tif]

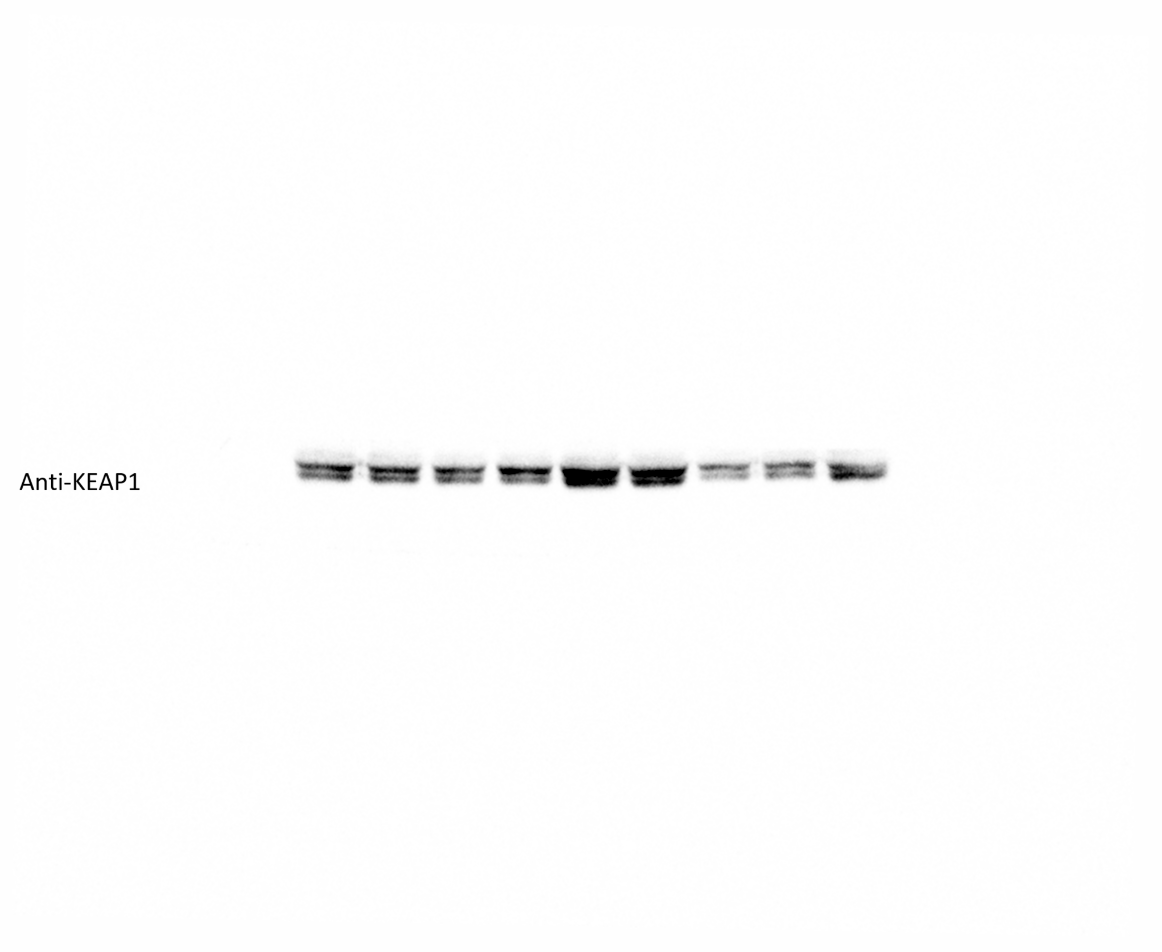

Supplement: S1 Raw images — (ZIP) [file pone.0316033.s003.zip › WB full blot band marker check v2/Figure6 KEAP1.tif]

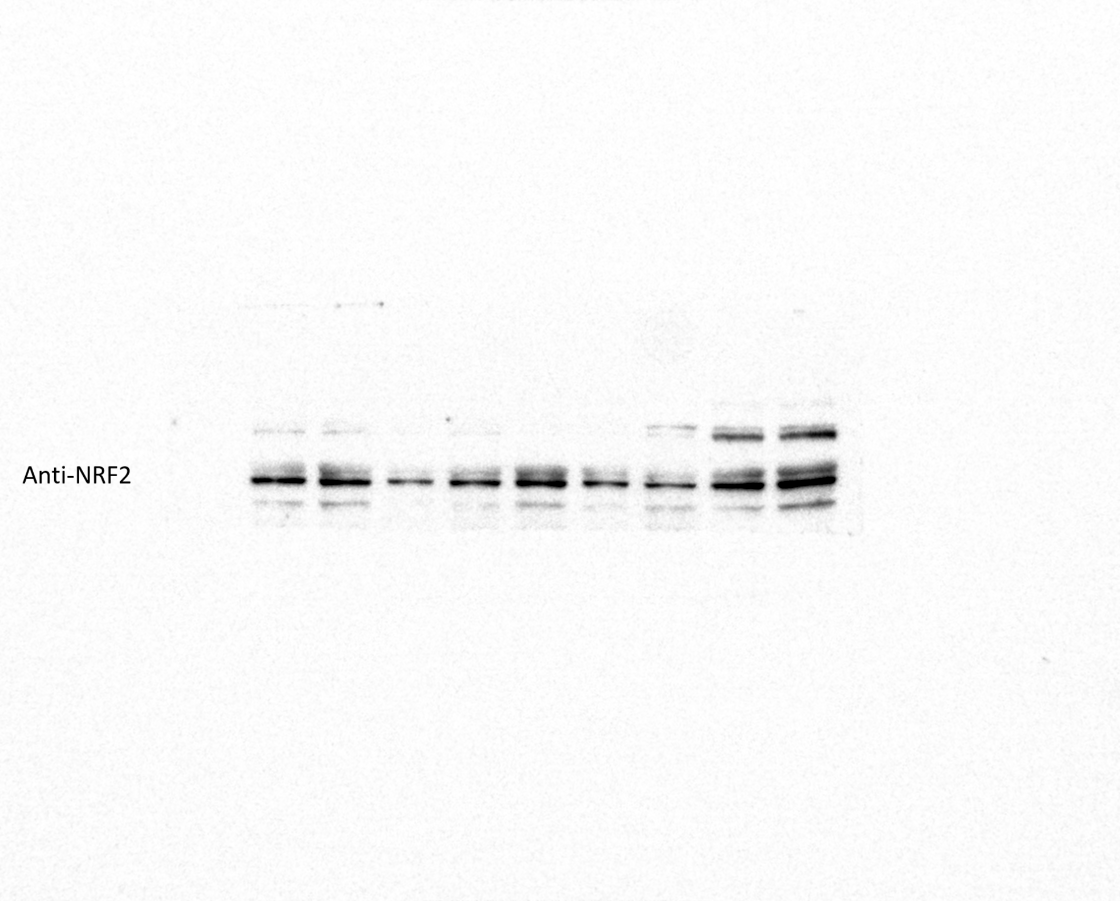

Supplement: S1 Raw images — (ZIP) [file pone.0316033.s003.zip › WB full blot band marker check v2/Figure6 NRF2.tif]
